# Climate change will lead to range shifts and genetic diversity losses of dung beetles in the Gobi Desert and Mongolian Steppe

**DOI:** 10.1038/s41598-024-66260-1

**Published:** 2024-07-08

**Authors:** Changseob Lim, Ji Hyoun Kang, Badamdorj Bayartogtokh, Yeon Jae Bae

**Affiliations:** 1 Ojeong Resilience Institute, Korea University, Seoul, Republic of Korea; 2 Korean Entomological Institute, Korea University, Seoul, Republic of Korea; 3 Department of Biology, School of Arts and Sciences, National University of Mongolia, Ulaanbaatar, Mongolia; 4 Division of Environmental Science and Ecological Engineering, Korea University, Seoul, Republic of Korea

**Keywords:** Desertification, Dryland, Ecological niche model, Mongolia, Scarabaeidae, Vulnerability, Biodiversity, Climate-change ecology

## Abstract

Desertification is known to be a major threat to biodiversity, yet our understanding of the consequent decline in biodiversity remains insufficient. Here, we predicted climate change-induced range shifts and genetic diversity losses in three model dung beetles: *Colobopterus erraticus*, *Cheironitis eumenes*, and *Gymnopleurus mopsus*, distributed across the Gobi Desert and Mongolian Steppe, areas known for desertification. Phylogeographic analyses of mitochondrial *COI* sequences and species distribution modeling, based on extensive field investigations spanning 14 years, were performed. Species confined to a single biome were predicted to contract and shift their distribution in response to climate change, whereas widespread species was predicted to expand even if affected by range shifts. We indicated that all species are expected to experience significant haplotype losses, yet the presence of high singleton frequencies and low genetic divergence across geographic configurations and lineages mitigate loss of genetic diversity. Notably, *Cheironitis eumenes*, a desert species with low genetic diversity, appears to be the most vulnerable to climate change due to the extensive degradation in the Gobi Desert. This is the first study to predict the response of insects to desertification in the Gobi Desert. Our findings highlight that dung beetles in the Gobi Desert and Mongolian Steppe might experience high rates of occupancy turnover and genetic loss, which could reshuffle the species composition.

## Introduction

Global climate change is a major threat to biodiversity and has been proposed to profoundly affect species distribution, genetic diversity, behavior, and fitness^1,2^. Species confronted with adverse climate change have been reported to exhibit diverse responses, including range-shifting by migration and persistence mediated by either phenotypic plasticity or local adaptation^1,3,4^. However, climate change that occurs at a rate surpassing species’ adaptive capacity can reduce population sizes and cause the extinction of local populations, inevitably eroding genetic diversity at the species level^3,5^.

Climate change models forecast an increase in global temperatures and disruption of precipitation patterns, which pose various challenges to ecosystems at regional scales. Desertification refers to the permanent degradation of land accompanied by changes in soil properties and precipitation. This process is a major climate change issue in arid, semi-arid, and sub-humid regions^6,7^. By the end of the twenty-first century, climate change could lead to an expansion of dryland area by 10% in total^7^. East Asia is one of the key areas that highly suffered from different levels of desertification along with the expansion of the Gobi Desert^8,9^. The Gobi Desert, the second largest rain shadow desert covering 1.3 million km^2^, stretches across the southern regions Mongolia as well as northern China^10^. So far, only a few studies have investigated and documented high rates of range shift on plant^11,12^ and mammals^13^ in the Gobi Desert and nearby sub-arid steppe region, and the impact of climate change on insects in this region has not yet been investigated.

Species distribution models (SDMs) are powerful tools for estimating the relative suitability of habitat for specific species^14^. As a result of the simplicity of modeling and the accessibility of fine-scale climatic data, SDMs have been implemented extensively to predict species’ current distribution and changes in species’ distribution that could arise in response to climate change^15,16^. However, SDM approaches, which are based on limited environmental data, have been criticized as oversimplistic since they do not account for evolutionary processes and biological features that affect species’ distribution^17,18^. For instance, integrating SDMs and phylogeographic information would provide a powerful framework for inferring the effects of future climate change on target organisms^5,19^.

Phylogeography may provide insight into the contemporary and future responses of species to environmental changes. Indeed, genetic structure can provide insight into a species’ evolutionary history and into the dynamics of contemporary gene flow among populations, and genetic diversity can be used to evaluate species’ evolutionary potential and resilience to environmental change^4,5^. In addition, populations that suffer from unsuitable habitats or limited connectivity often harbor low genetic diversity as a result of bottlenecks or inbreeding depression and are, as a result, highly vulnerable to environmental change^20^. Therefore, investigating the genetic diversity and structure of populations across species’ distribution ranges is critical to evaluating the vulnerability and adaptive capacity of species to environmental change^5,21^.

Dung beetles are key organisms in terrestrial ecosystems because they contribute to various ecosystem processes, including nutrient cycling, parasite suppression, and secondary seed dispersal^22^. The group has been widely used as a bioindicator of biodiversity and ecosystem health because of their ecological importance, sensitivity to environmental factors, and established standardized sampling methods^23,24^. Serious concerns exist regarding the global decline in dung beetle diversity, and empirical data show that populations of many dung beetle species have significantly declined in response to habitat fragmentation^25^ and climate change^26,27^.

Mongolia spans a vast plateau with wide steppe and desert regions. More than 67 million livestock, including horses, cattle, sheep, goats, camels, and yaks, are farmed across the Gobi Desert and Mongolian Steppe, mostly through nomadic herding (National Statistics Office of Mongolia; https://www.en.nso.mn/, accessed on April 08, 2022). This environment, abundant in food resources throughout the country, dung beetles are essential for maintaining the ecosystem health. To date, 78 dung beetle species have been documented in Mongolia^28^. Research on the conservation of dung beetles in Mongolia has been primarily confined to a few national reserves^29,30^. However, Mongolia is a particularly well-suited region for investigating the effects of desertification through climate change on organisms. Encompassing two distinct climate regions and less affected by other anthropogenic disturbances such as habitat fragmentation, and chemical treatments, the country act as a natural laboratory to pinpoint and understand the direct implications of climate change.

Accordingly, this study explored how future climate change-induced range shifts and the loss of genetic diversity may differ among dung beetles in desert and sub-arid steppe region by incorporating SDMs and phylogeographic analyses. To achieve this, we investigated the genetic diversity, population structure, demographic history, and distribution of three model species from the Gobi Desert and Mongolian Steppe in Mongolia. We then predicted their future distribution and genetic diversity under climate change scenarios.

## Results

### Genetic diversity and haplotype network analyses

Mitochondrial *COI* sequences were generated from 173, 152, and 273 specimens of *C. erraticus* (12 locations)*, C. eumenes* (10 locations), and *G. mopsus* (10 locations)*,* respectively (Table 1). The *C. erraticus* sequences (836 bp) yielded 29 polymorphisms, including 18 parsimony-informative sites and 44 haplotypes (36 singletons; 81.8%). Meanwhile, the *C. eumenes* sequences (789 bp) yielded 29 polymorphisms, including 16 parsimony-informative sites and 26 haplotypes (18 singletons, 69.2%), and the *G. mopsus* sequences (658 bp) yielded 81 polymorphisms, including 51 parsimony-informative sites and 135 haplotypes (111 singletons; 82.2%).

**Table 1:** Localities and genetic diversity indices for sampled populations of three species of dung beetles in Mongolia.

Species	Sites	Regions	Coordinates	Number of individuals (*N*)	Number of Haplotypes (*Nh*)	Allelic richness (*Ar*)	Haplotype diversity (*Hd*) (SD)	Nucleotide diversity (π) (SD)	Tajima’s *D*	Fu’s *Fs*
*Colobopterus erraticus*	CER1402	Selenge Aimag	49° 38′ 46.26′′ N 106° 01′ 42.64′′ E	14	6	1.778	0.7363 (0.1092)	0.0013 (0.0010)	− 1.1150	**− 2.7635**
	CER1502	Töv Aimag	47° 26′ 56.36′′ N 106° 46′ 42.80′′ E	5	3	1.600	0.7000 (0.2184)	0.0022 (0.0017)	− 0.4102	0.4690
	CER16EX2	Selenge Aimag	50° 05′ 58.4′′ N 106° 12′ 47.7′′ E	4	3		0.8333 (0.2224)	0.0026 (0.0021)	− 0.0650	0.6080
	CER1621	Bayankhongor Aimag	46° 23′ 15.85′′ N 100° 48′ 48.63′′ E	19	9	2.196	0.8421 (0.0670)	0.0049 (0.0029)	− 0.4079	− 0.8753
	CER1626	Arkhangai Aimag	47° 23′ 07.53′′ N 102° 12′ 57.64′′ E	8	5	2.214	0.8571 (0.1083)	0.0021 (0.0015)	− 0.5034	− 1.4953
	CER1808	Zavkhan Aimag	47° 41′ 22.65′′ N 97° 15′ 30.93′′ E	18	13	2.770	0.9608 (0.0301)	0.0084 (0.0047)	1.3475	0.9310
	CER1822	Khovd Aimag	48° 00′ 59.70′′ N 91° 37′ 15.25′′ E	20	6	1.842	0.0000 (0.0624)	0.0016 (0.0002)	0.4159	− 1.5046
	CER1005	Khövsgöl Aimag	50° 32′ 15.6′′ N 101° 30′ 55.6′′ E	1	1		1.0000 (0.0000)	0.0000 (0.0000)	0.0000	0.0000
	CER2208	Arkhangai Aimag	47° 35′ 17.73′′ N 101° 12′ 51.48′′ E	22	11	2.374	0.8831 (0.0471)	0.0053 (0.0030)	0.2825	− 1.7727
	CER2213	Arkhangai Aimag	48° 23′ 44.08′′ N 101° 18′ 46.40′′ E	16	9	2.406	0.8917 (0.0543)	0.0028 (0.0018)	− 1.1568	**− 3.5835**
	CER2218	Khövsgöl Aimag	49° 35′ 31.79′′ N 101° 59′ 14.78′′ E	23	6	1.816	0.7668 (0.0489)	0.0015 (0.0011)	− 0.2460	− 1.3902
	CER2222	Bulgan Aimag	48° 57′ 47.33′′ N 102° 47′ 27.31′′ E	23	7	1.898	0.7826 (0.0533)	0.0015 (0.0011)	− 1.0794	**− 2.4441**
	Total			173	44		0.8370 (0.0180)	0.0037 (0.0004)	− 1.1127	**− 26.2318**
*Cheironitis eumenes*	CEU1609	Umnugobi Aimag	44° 09′ 38.65′′ N 104° 05′ 29.37′′ E	22	7	1.729	0.5974 (0.1184)	0.0015 (0.0011)	**− 1.7885**	**− 2.8194**
	CEU1713	Dornogobi Aimag	45° 13′ 28.06′′ N 110° 8′ 46.33′′ E	24	5	1.431	0.5400 (0.1089)	0.0015 (0.0011)	− 1.4774	− 0.5116
	CEU1415	Dornogobi Aimag	46° 0′ 11.87′′ N 108° 50′ 7.61′′ E	5	4	3.000	0.9000 (0.1610)	0.0030 (0.0023)	− 1.1455	− 0.7012
	CEU1706	Dornogobi Aimag	45° 49′ 9.25′′ N 109° 18′ 11.57′′ E	3	3		1.0000 (0.2722)	0.0068 (0.0056)	0.0000	0.4576
	CEU1607	Umnugobi Aimag	45° 49′ 9.25′′ N 109° 18′ 11.57′′ E	2	1		0.0000 (0.0000)	0.0000 (0.0000)	0.0000	0.0000
	CEU1608	Umnugobi Aimag	44° 10′ 41.30′′ N 104° 22′ 13.87′′ E	5	1	0.000	0.0000 (0.0000)	0.0000 (0.0000)	0.0000	0.0000
	CEU22A4	Umnugobi Aimag	44° 21′ 18.26′′ N 105° 15′ 26.49′′ E	24	9	2.172	0.7065 (0.0992))	0.0019 (0.0013)	− 1.4720	**− 4.0130**
	CEU22A6	Umnugobi Aimag	43° 56′ 56.6′′ N 104° 57′ 41.0′′ E	28	6	1.281	0.4815 (0.1083)	0.0014 (0.0010)	**− 1.8656**	− 1.5132
	CEU22A7	Umnugobi Aimag	43° 54′ 51.3′′ N 104° 55′ 14.4′′ E	21	6	1.646	0.6000 (0.1104)	0.0009 (0.0007)	− 1.4856	**− 3.3056**
	CEU22A8	Umnugobi Aimag	43° 41′ 12.7′′ N 104° 39′ 42.2′′ E	18	5	1.111	0.4052 (0.1428)	0.0013 (0.0010)	**− 2.1893**	− 1.2567
	Total			152	26		0.5660 (0.0480)	0.0016 (0.0002)	**− 2.2122**	**− 25.4402**
*Gymnopleurus mopsus*	GM1403	Selenge Aimag	49° 45′ 25.43′′ N 106° 9′ 58.01′′ E	34	20	6.925	0.9340 (0.0301)	0.0082 (0.0045)	− 1.32765	**− 7.48445**
	GM1417	Dornogobi Aimag	45° 09′ 19.52′′ N 109° 58′ 0.28′′ E	34	28	8.398	0.9857 (0.0115)	0.0081 (0.0045)	− 1.4744	**− 22.7255**
	GM1510	Umnugobi Aimag	44° 14′ 16.51′′ N 106° 53′ 59.43′′ E	12	11	8.318	0.9848 (0.0403)	0.0082 (0.0048)	− 1.30233	**− 5.19347**
	GM1604	Dundgobi Aimag	44° 54′ 47.99′′ N 105° 32′ 37.74′′ E	28	28	9.000	1.0000 (0.0101)	0.0091 (0.0050)	− 1.49167	**− 25.26433**
	GM1605	Dundgobi Aimag	43° 46′ 28.95′′ N 104° 47′ 29.96′′ E	32	29	8.661	0.9919 (0.0110)	0.0081 (0.0045)	− 1.33654	**− 25.38005**
	GM1611	Umnugobi Aimag	44° 22′ 33.54′′ N 104° 02′ 32.60′′ E	28	27	8.881	0.9974 (0.0104)	0.0091 (0.0050)	**− 1.50788**	**− 24.92409**
	GM1628	Orkhon Aimag	47° 21′ 02.14′′ N 103° 44′ 06.94′′ E	34	19	6.722	0.9376 (0.0213)	0.0091 (0.0049)	− 0.74768	− 3.40479
	GM1630	Töv Aimag	47° 44′ 27.8′′ N 105° 51′ 58.7′′ E	37	10	5.289	0.8889 (0.0226)	0.0091 (0.0049)	0.28248	1.79525
	GM162b	Gobisumber Aimag	46° 06′ 26.39′′ N 108° 43′ 20.88′′ E	11	11	9.000	1.0000 (0.0388)	0.0098 (0.0057)	− 0.82427	**− 5.8677**
	GM2217	Khövsgöl Aimag	49° 27′ 32.61′′ N 101° 32′ 20.78′′ E	23	7	4.077	0.7826 (0.0717)	0.0072 (0.0041)	0.30558	1.85054
	Total			273	135		0.9717 (0.0044)	0.0088 (0.0003)	**− 1.6712**	**− 24.7955**

Significant values (*P* < 0.05, neutrality test) are indicated in bold.

Estimates of haplotype diversity for populations represented by at least five specimens (N ≥ 5) ranged from 0.7000 to 0.9608 (mean = 0.8370), from 0.0000 to 0.9000 (mean = 0.5660), and from 0.7826 to 1.0000 (mean = 0.9717) for *C. erraticus*, *C. eumenes*, and *G. mopsus*, respectively (Table 1). Meanwhile, nucleotide diversity estimates ranged from 0.0013 to 0.0084 (mean = 0.0037), from 0.0000 to 0.0030 (mean = 0.0016), and from 0.0072 to 0.0098 (mean = 0.0088) for *C. erraticus*, *C. eumenes*, and *G. mopsus*, respectively (Table 1). Most of the *C. erraticus* and *C. eumenes* localities yielded relatively low allelic richness (*Ar*), ranging from 1.600 to 2.770 (*C. erraticus*) and from 0.000 to 3.000 (*C. eumenes*). In contrast, the *G. mopsus* localities yielded relatively high allelic richness, ranging from 4.077 to 9.000.

**Figure 1:**
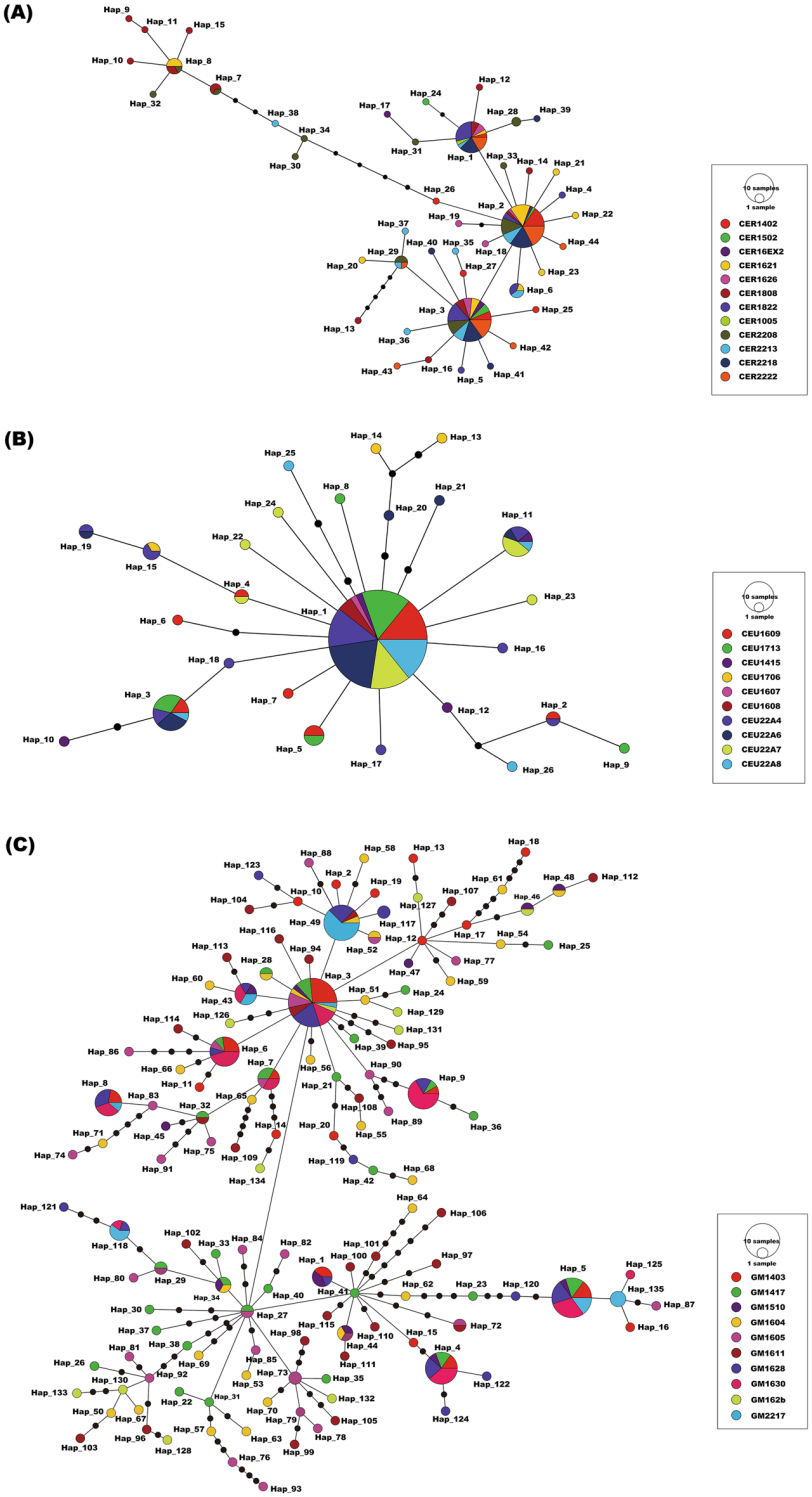
Haplotype networks of *COI* sequences of three model species. (**A**) *Colobopterus erraticus*, (**B**) *Cheironitis eumenes*, (**C**) *Gymnopleurus mopsus*.

The haplotype networks of three species exhibited different topologies (Fig. 1). In the *C. erraticus* haplotype network*,* three dominant haplotypes (H1, H2, and H3), each one mutation step apart, accounted for 13.9% (H1, N = 24), 26.6% (H2, N = 46), and 27.2% (H3, N = 47), respectively. Haplotypes from CER1808, CER1621, and CER2213, clustered into haplogroups with approximately 10 mutational steps of H2 (Fig. 1A). In the *C. eumenes* haplotype network*,* the most dominant haplotype (H1) accounted for 65.1% (N = 99) of specimens and was located at the most internal position in the network, presumably to be an ancestral haplotype (Fig. 1B). In the *G. mopsus* haplotype network, the most dominant haplotype (H3) accounted for 11.0% (N = 30) of specimens and was located at an internal position in the network (Fig. 1C). This haplotype was detected in specimens from all the sampled localities, which indicated its extensive distribution.

### Population structure and demographic history analyses

Populations of *C. erraticus* and *G. mopsus* exhibited significant pairwise genetic differentiation, whereas those of *C. eumenes* did not*.* The pairwise differences (*F*_ST_) of *C. erraticus* populations ranged from − 0.066 to 0.367 (Supplementary Information, Table S1). However, the genetic differentiation between CER1808 and the other three sites was significant, which indicate that the local population is genetically isolated from the others. Meanwhile, the pairwise differences (*F*_ST_) of *C. eumenes* ranged from − 0.087 to 0.081 (Table S2), with all *F*_ST_ values indicating low levels of genetic differentiation. Finally, the pairwise differences (*F*_ST_) of *G. mopsus* populations ranged from − 0.014 to 0.078 (Table S3). As in *C. erraticus*, most of the *F*_ST_ values were relatively small, indicating a low inter-population differentiation level. Only four of the 45 comparisons among the 10 populations indicated significant differentiation.

In *C. erraticus*, AMOVA indicated significant spatial genetic differentiation between the Khangai Mountains group and other populations (Table 2). In *G. mopsus*, AMOVA indicated that significant spatial genetic structuring occurred under climate and geography, as reported by previous study^31^. However, no spatial genetic structuring was observed in the geographical groupings of *C. eumenes*.

**Table 2:** Analysis of molecular variance (AMOVA) for sampled populations of three dung beetle species in Mongolia.

Species	Hypothesis	d.f	Percent of Variation	F-statistics	P value
*Colobopterus erraticus*	Two groups (Khangai, Others)	1	19.13	0.19132	**0.00782**
		8	2.48	0.03073	0.06354
		158	78.38	0.21616	**0.000001**
	Three groups (N, C, W)	2	− 0.3	− 0.00299	0.39785
		7	13.78	0.13742	**0.000001**
		158	86.52	0.13483	**0.000001**
*Cheironitis eumenes*	Two groups (S, SE)	1	0.86	0.00856	0.261
		6	− 0.18	− 0.00182	0.49462
		139	99.32	0.00675	0.4086
*Gymnopleurus mopsus*	Two groups (Steppe, Desert)	1	2.5	0.025	**0.00978**
		8	1.19	0.01223	**0.02248**
		263	96.31	0.03692	**0.000001**
	Three groups (N, C, S)	2	2.02	0.02025	**0.0088**
		7	1.22	0.01249	**0.02542**
		263	96.75	0.03248	**0.000001**
	Four groups (NC, NW, S, SE)	3	2.34	0.02344	**0.000001**
		6	0.69	0.0071	0.15738
		263	96.96	0.03037	**0.000001**

Significant values (*P* < 0.05) are indicated in bold.

The evaluation of the population expansion in *C. eumenes* and *G. mopsus* yielded significantly negative values when all populations were pooled in a single dataset (Table 1). Evaluation of *C. erraticus* yielded negative values for both indices (Tajima’s *D* = − 1.1127, *P* = 0.1230; Fu’s *Fs* =  − 26.2318, *P* < 0.0001), and only Fu’s *Fs* was significant. A multimodal mismatch distribution of pairwise differences was observed for the *C. erraticus* populations, with significant statistics for both the sum of squared deviations (SSD) and raggedness index values (SSD = 0.0151, *P* < 0.0001, raggedness index = 0.0653, *P* < 0.0001), which indicated a constant population size under the neutral model (Supplementary Information, Fig. S1). A unimodal mismatch distribution was observed for *C. eumenes* (skewed) and *G. mopsus*. However, the SSD and raggedness index values were non-significant (*C. eumenes*: SSD = 0.0078, *P* = 0.66; raggedness index = 0.0629, *P* = 0.54; *G. mopsus*: SSD = 0.0005, *P* = 0.92; raggedness index = 0.0053, *P* = 0.91), which indicated historical population expansion (Fig. S1).

### Species distribution modeling

**Figure 2:**
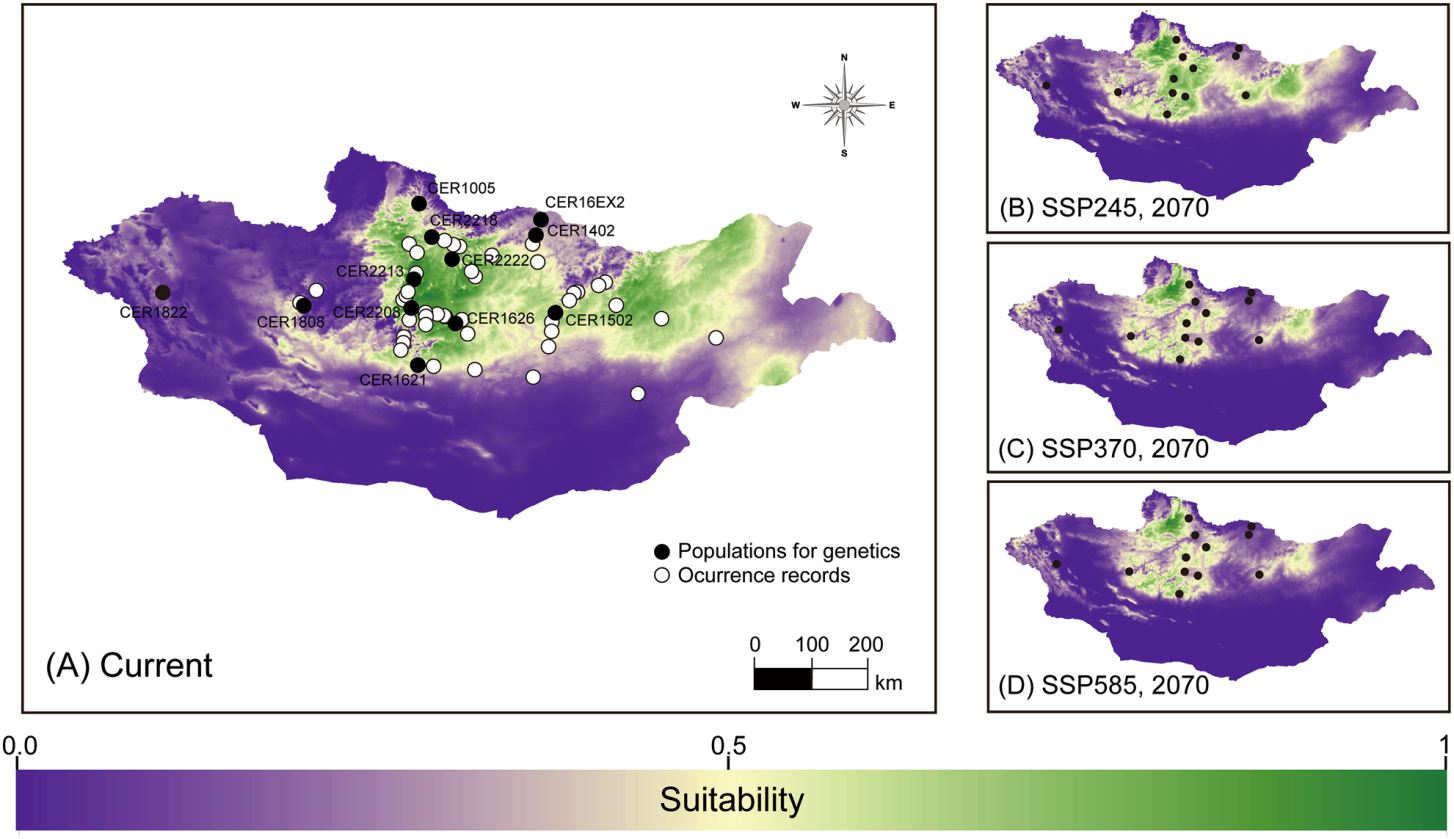
Distribution and environmental suitability predictions of *Colobopterus erraticus.* (**A**) Current species distribution model with the occurrence records (white dots), and sites for population genetics (black dots); (**B**–**D**) Predicted future distributions in 2070 under the SSP245, SSP370, and SSP585 scenarios, respectively. The map of distribution model was modified using ArcGIS 10.8 (www.esri.com)^67^.

**Figure 3:**
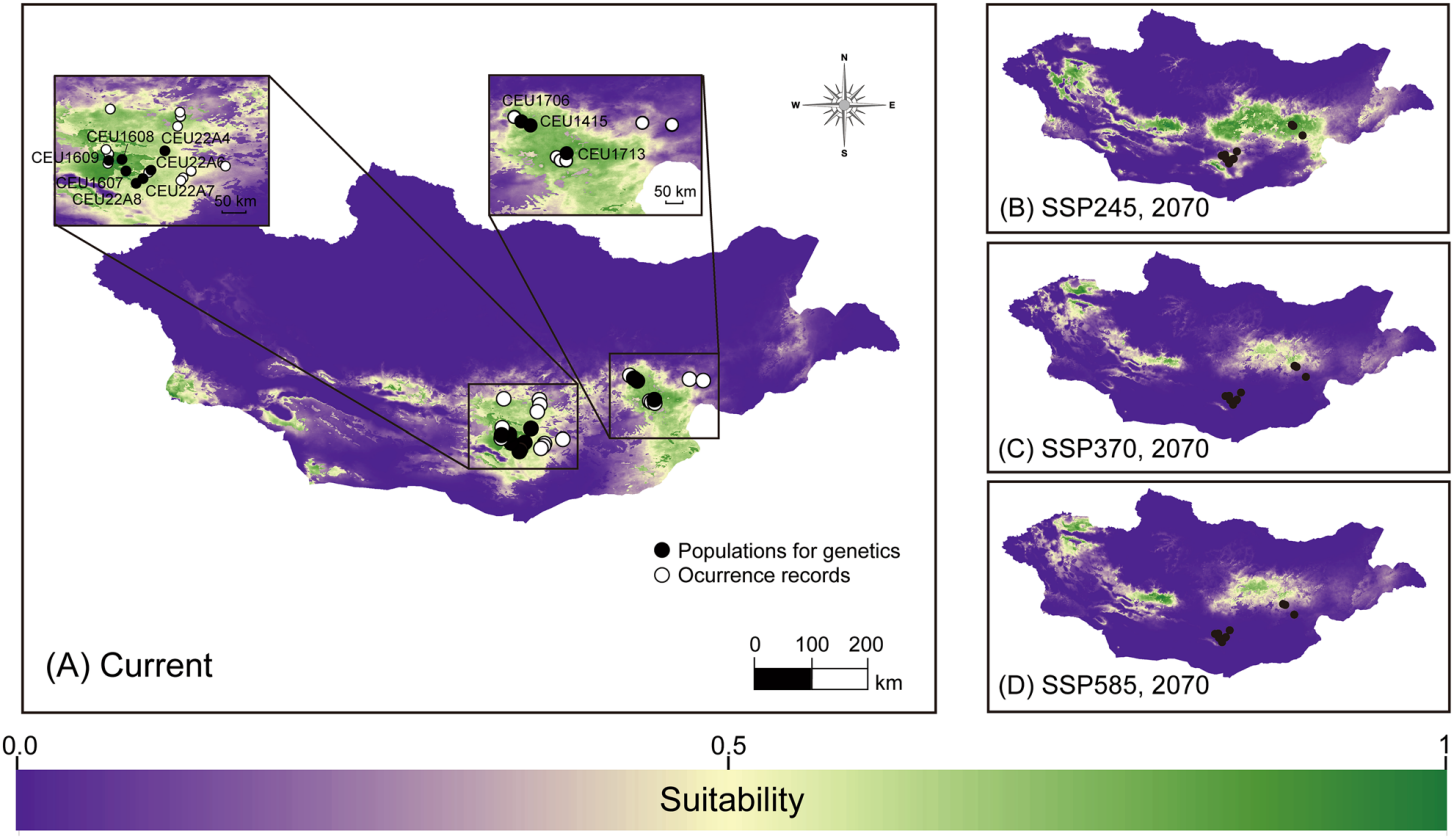
Distribution and environmental suitability predictions of *Cheironitis eumenes.* (**A**) Current species distribution model with the occurrence records (white dots), and sites for population genetics (black dots); (**B**–**D**) Predicted future distributions in 2070 under the SSP245, SSP370, and SSP585 scenarios, respectively. The map of distribution model was modified using ArcGIS 10.8 (www.esri.com)^67^.

**Figure 4:**
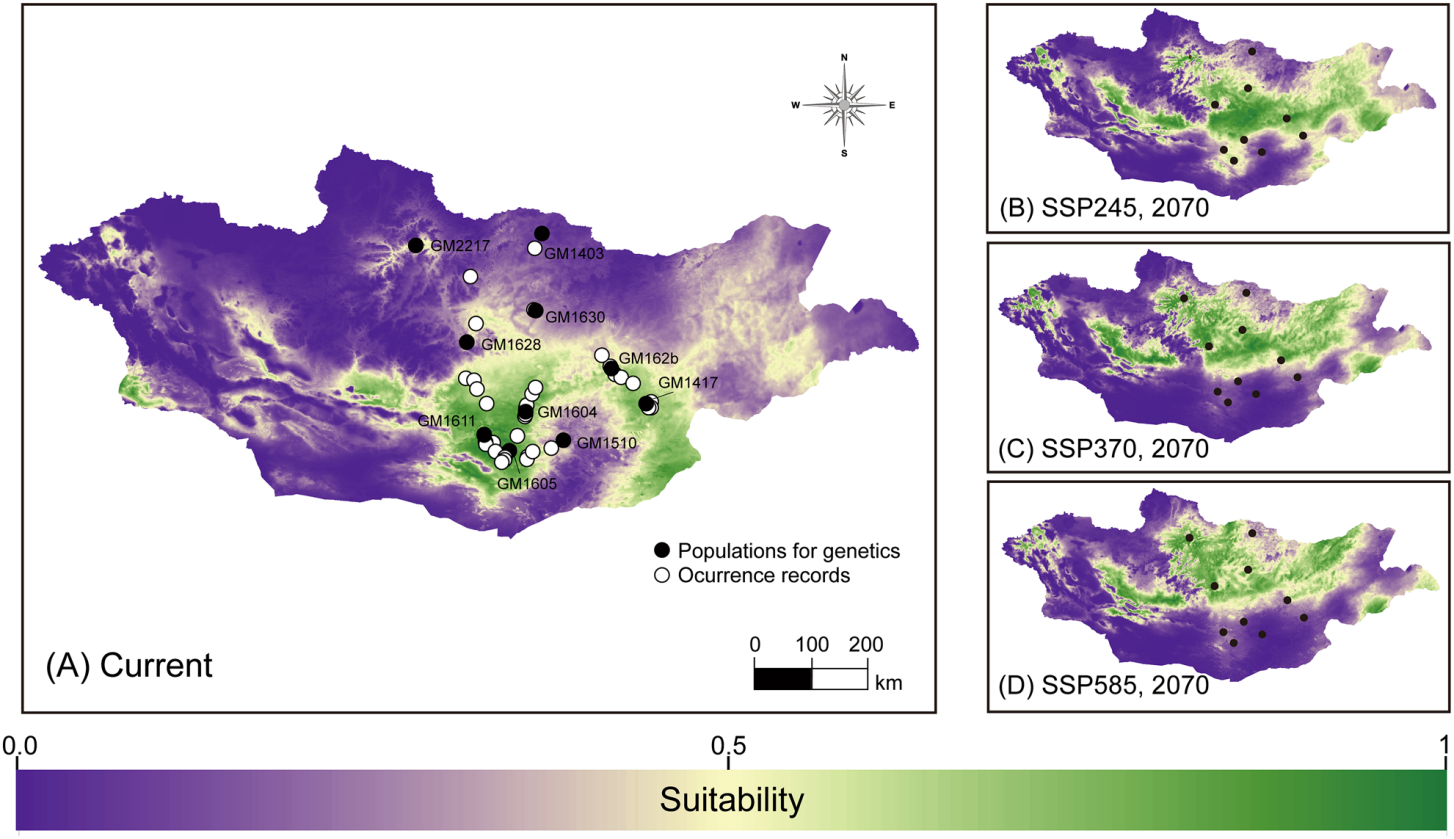
Distribution and environmental suitability predictions of *Gymnopleurus mopsus.* (**A**) Current species distribution model with the occurrence records (white dots), and sites for population genetics (black dots); (B–D) Predicted future distributions in 2070 under the SSP245, SSP370, and SSP585 scenarios, respectively. The map of distribution model was modified using ArcGIS 10.8 (www.esri.com)^67^.

The optimal SDMs for each species yielded satisfactory predictive performance (*C. erraticus*: RM = 1, FC = LQ, AUC_test_ = 0.830, AUC_diff_ = 0.077, OR_10_ = 0.140, ΔAICc = 0; *C. eumenes*: RM = 0.5, FC = LQ, AUC_test_ = 0.953, AUC_diff_ = 0.033, OR10 = 0.100, ΔAICc = 0; *G. mopsus*: RM = 1.5, FC = LQHP, AUC_test_ = 0.860, AUC_diff_ = 0.071, OR10 = 0.105, ΔAICc = 17.350; Fig. S2). The models predicted that *C. erraticus* and *C. eumenes* were distributed in the northeastern steppe and southern desert, respectively, and predicted that *G. mopsus* was distributed among both steppe and desert biomes across central Mongolia (Figs. 2, 3, and 4, S3–S5). Presence–absence binary maps were created using 10% training logistic thresholds for each species (thresholds: *C. erraticus*, 0.2864; *C. eumenes*, 0.3427; and *G. mopsus*, 0.2931).

### Predicting distribution and genetic diversity under climate change scenarios

Estimations of future *C. erraticus* distribution indicated a westward shift. The northeastern area of the species’ distribution contracted and shifted among the various time points and climate scenarios (Fig. 2). Under all climate scenarios, the distribution of *C. erraticus* exhibited a net reduction in area, ranging from − 18.55 to − 46.26% and from − 31.80 to − 63.23% in 2050 and 2070, respectively (Table 3). In contrast, the future projections of *C. eumenes* revealed a severe reduction in the southern desert region and a northward shift to central steppe region (Fig. 3). The distribution of *C. eumenes* was projected to either increase or decrease, with predicted changes in area ranging from − 79.01 to 19.98% and from − 28.02 to 58.42% in 2050 and 2070, respectively (Table 3). For *G. mopsus*, the species’ distribution was predicted to shift northward, with a net expansion in the northern steppe region (Fig. 4). Under all climate scenarios, the distribution of *G. mopsus* exhibited a net expansion in the area, ranging from 27.23 to 33.49% and from 21.63 to 43.44% in 2050 and 2070, respectively (Table 3).

**Table 3:** Predicted future genetic diversity and distribution for sampled populations of three dung beetle species in Mongolia under current conditions and future climate change scenarios.

Species	Scenarios/Time period	Haplotype retained (%)	Haplotype diversity (*Hd*)	Nucleotide diversity (π)	Change in distribution area from Current (%)
*Colobopterus erraticus*	Current		0.837	0.004	
	SSP245/2050	86.36	0.848	0.004	− 18.55
	SSP245/2070	86.36	0.848	0.004	− 31.80
	SSP370/2050	79.55	0.865	0.005	− 37.70
	SSP370/2070	65.91	0.862	0.005	− 55.83
	SSP585/2050	79.55	0.865	0.005	− 46.26
	SSP585/2070	45.55	0.827	0.003	− 63.23
*Cheironitis eumenes*	Current		0.566	0.002	
	SSP245/2050	57.69	0.584	0.002	+ 8.49
	SSP245/2070	80.77	0.562	0.002	+ 58.42
	SSP370/2050	80.77	0.562	0.002	+ 19.98
	SSP370/2070	11.54	1.000	0.007	− 36.80
	SSP585/2050	26.92	0.964	0.005	− 79.01
	SSP585/2070	0	0	0	− 28.02
*Gymnopleurus mopsus*	Current		0.971	0.009	
	SSP245/2050	89.63	0.975	0.009	+ 29.46
	SSP245/2070	89.63	0.975	0.009	+ 43.44
	SSP370/2050	89.63	0.975	0.009	+ 33.49
	SSP370/2070	31.11	0.937	0.009	+ 21.63
	SSP585/2050	56.30	0.962	0.009	+ 27.23
	SSP585/2070	31.11	0.937	0.009	+ 21.89

The predicted loss of future genetic diversity in the overall population varied based on the climatic niche and phylogeography of each species. For example, six of the 12 *C. erraticus* populations were predicted to be affected by climate change (Table S4). The CER1402, CER16EX2, and CER1822 populations were predicted to be extirpated in every scenario, whereas CER1808 (currently exhibiting low suitability) increased in suitability under most of the future climatic scenarios (all but SSP585 in 2070). In addition, 24 (54.6%) of the 44 *C. erraticus* haplotypes were estimated to be affected by at least one climate change scenario, and six were predicted to be completely lost in response to climate change (Fig. S6). For *C. eumenes*, all populations were predicted to be affected by climate change (Table S4). The CEU22A4 and CEU22A8 populations were predicted to be extirpated in every scenario, and under the most severe scenario, local extinction was predicted for all populations by 2070. In addition, five (19.2%) of the *C. eumenes* 26 haplotypes were predicted to be lost in response to climate change (Fig. S7). For *G. mopsus*, six of the 10 populations were predicted to be affected by climate change (Table S4). The GM1510 population was predicted to be extirpated in every scenario, whereas GM1403 (currently exhibiting low suitability) was predicted to increase in suitability. In addition, 107 (79.3%) of the 135 *G. mopsus* haplotypes were predicted to be affected by at least one climate change scenario, and two haplotypes were predicted to be lost (Fig. S8). However, despite the predicted losses in the number of haplotypes, none of the three species were expected to undergo large reductions in haplotype or nucleotide diversity (Table 3). Similarly, there was no evident loss of phylogenetic diversity (e.g., loss of clades) in the phylogenetic tree of each species. Instead, the missing haplotypes were scattered throughout the tree.

## Discussion

This study is the first to model the effects of climate change on insects from the Gobi Desert and Mongolian Steppe region. Given the serious concerns about desertification in the Gobi Desert and adjacent regions^7,8^, there have been few studies investigating its influences on organisms there^11–13^. This issue fundamentally stems from the lack of accumulated data in this region. Our results on the distribution and genetic diversity of Mongolian dung beetles can be valuable data for future studies on global-scale desertification as well as for the responses of organisms to desertification in the Gobi Desert.

Genetic diversity and structure can be used as indicators of the tolerance and adaptive potential of a population to environmental stress^32^. In this study, *C. erraticus* and *G. mopsus* exhibited high genetic diversity with high singleton frequency, which could reflect the species’ adaptive potential. In contrast, the desert-dwelling *C. eumenes* exhibited moderate genetic diversity, with a single haplotype accounting for 65% of specimens, which could indicate that the adaptive potential of this species is lower than that of either *C. erraticus* or *G. mopsus*.

Most of the *F*_ST_ values among the populations of the three species were relatively small, suggesting low levels of interpopulation genetic differentiation, which could be related to contemporary gene flow or demographic events such as bottlenecks, recent divergence, or selection. For example, there were no significant *F*_ST_ values for distant populations, *C. erraticus* populations at CER1822 and CER1502 (over 1100 km)*, C. eumenes* populations at CEU1609 and CEU1713 (over 450 km), and *G. mopsus* populations at GM1417 and GM2207 (over 790 km; Tables S1–S3). Notably, significant differences were observed between the Khangai Mountains and other populations of *C. erraticus* (Table 2). These differences could be derived from historical demographic events involving geographic barriers, for instance, the founder effect or selection on historically small, isolated populations from genetically diverse population in the past^33^. This phenomenon was observed only in the Khangai Mountains populations, whereas the Altai Mountains population (CER1822) did not exhibit significant differences from the other populations (except for CER1808). The genetic structures in both geographic and climatic group for *G. mopsus* coincided with those reported by previous study^31^.

The demographic histories of the three dung beetle species were observed to differ. The haplotype networks of all three species showed major haplotypes surrounded by many unique haplotypes. For *C. eumenes*, a single major haplotype was identified, while multiple major haplotypes were observed in the other two species. Our demographic history analyses indicated that *C. eumenes* and *G. mopsus* have undergone recent rapid expansion. A previous study proposed the domestication-driven expansion hypothesis to explain this recent expansion of Mongolian dung beetles^31^. However, Tajima’s D analysis and the mismatched distribution of the overall population revealed that *C. erraticus* maintained a constant-sized population rather than undergoing a recent expansion. One plausible explanation for this result could be the difference in food ranges among the three species. *C. eumenes* and *G. mopsus* prefer the feces of large herbivores, primarily livestock, whereas *C. erraticus* has a broader food range that includes the feces of small mammals. The presence of *C. erraticus* inside the burrows of rodents has been reported, indicating that the species can utilize the dung or residues found within burrows^34^. Therefore, *C. erraticus* may have been less affected by domestication events than the other two species. Another hypothesis is that the demographic histories of dung beetles in Mongolia were influenced more by late Quaternary climate change than by domestication events. Several previous studies have reported the influence of the late Quaternary climate oscillation, usually the last glacial maximum, on the population demography of species in Mongolia^35,36^.

The SDMs used in this study suggest that the distributions of dung beetles inhabiting a single biome type will contract and shift substantially in response to climate change. In contrast, the distributions of species inhabiting multiple biomes will expand, even if affected by range shifts. These trends are consistent with the idea that generalists and specialists respond differently to rapid environmental change. Species with broad tolerance exhibit range expansion, whereas species with restricted tolerance undergo range contraction^37,38^.

We revealed that the number of haplotypes would decrease following the extinction of local populations of all three species (Table 3). Under the most severe climate scenario (SSP585), *C. erraticus* and *G. mopsus* were predicted to experience haplotype losses of 54.5 and 68.9%, respectively, by 2070. Meanwhile, *C. eumenes* was predicted to have lost all extant haplotypes from the 10 sites investigated in this study. A reduction in the absolute number of haplotypes within the overall gene pool of a species is likely to reduce its evolutionary potential and resilience. However, despite reduced haplotype richness, the dung beetles modeled in this study were predicted to maintain genetic diversity (Table 3). These unexpected results may be attributed to the low mtDNA divergence of the species in both geographic configurations and lineages*.* In the phylogenetic trees of these species, unique haplotypes were scattered without geographic configuration or deep phylogenetic divergence. Therefore, haplotype loss due to local extinction would be significant under climate change scenarios, while low genetic divergence and a high singleton frequency could buffer the effects of climate change on genetic diversity and phylogeographic structure. Similar predictions have been reported by previous studies^19,39^.

Our predictions of future genetic diversity and distribution should be interpreted cautiously. First, the study could not address the extent to which dispersal might mitigate genetic diversity loss, and it was conservatively assumed that dispersal was restricted. In this study, local populations were assumed to disappear as habitats became climatically unsuitable, even when they were close to areas projected to persist or become climatically suitable. This assumption can enhance the prediction of potential genetic diversity loss, thereby contributing to effective conservation planning. However, it can reduce the prediction accuracy. For instance, the range of each species could shift due to enhanced fitness in locations at the leading edge and reduced fitness in locations at the trailing edge. This process tends to lead to a gradient in genetic diversity that increases toward the leading edge^32,40^. Second, the binary decision of presence or absence in the model neglected phenotypic plasticity and local adaptation. Several studies have investigated the local adaptation and phenotypic plasticity of dung beetles in terms of climatic differences. For example, Lim et al. (2020) reported variations in the morphology (body shape and size) of *G. mopsus* from steppe and desert in Mongolia^41^. Furthermore, Cuesta et al.^42^ reported the phenological plasticity of Iberian dung beetles, and Macagno et al. (2018) reported the behavioral plasticity of the model species *Onthophagus taurus*, which was observed to mitigate the negative effects of temperature increases^43^. This study indicates that *C. erraticus* and *G. mopsus* inhabit climatically unsuitable localities (*C. erraticus*: CER1822 and CER1005; *G. mopsus*: GM1403, GM1510, and GM1630) with relatively high genetic diversity (except for CER1005, which included only one individual), which might suggest that, in Mongolia, the response of dung beetles to climatic stress involves either local adaptation or phenotypic plasticity.

Researchers have recently attempted to incorporate evolutionary factors into SDMs^44,45^. For example, Benito-Garzόn et al.^46^ described a newly developed SDM that can incorporate fitness-related traits to estimate the sensitivity of species to environmental change. Abreu‐Jardim et al.^39^ forecasted the effects of climate change on the phylogeographic diversity of species. Nevertheless, such efforts remain in the early stages of development and have been applied to a few well-documented species. Determining the actual levels of genetic parameters required for responding to environmental change is challenging. Nonetheless, it’s essential to conserve a wide range of genotypes across both phylogeny and geography to retain a meaningful subset of this diversity^21^. Our results highlight that the spatial arrangement of haplotypes and lineages, reflecting the distinct phylogeographic histories of each species, offers valuable insights into their resistance against potential genetic diversity loss owing to climate change.

**Figure 5:**
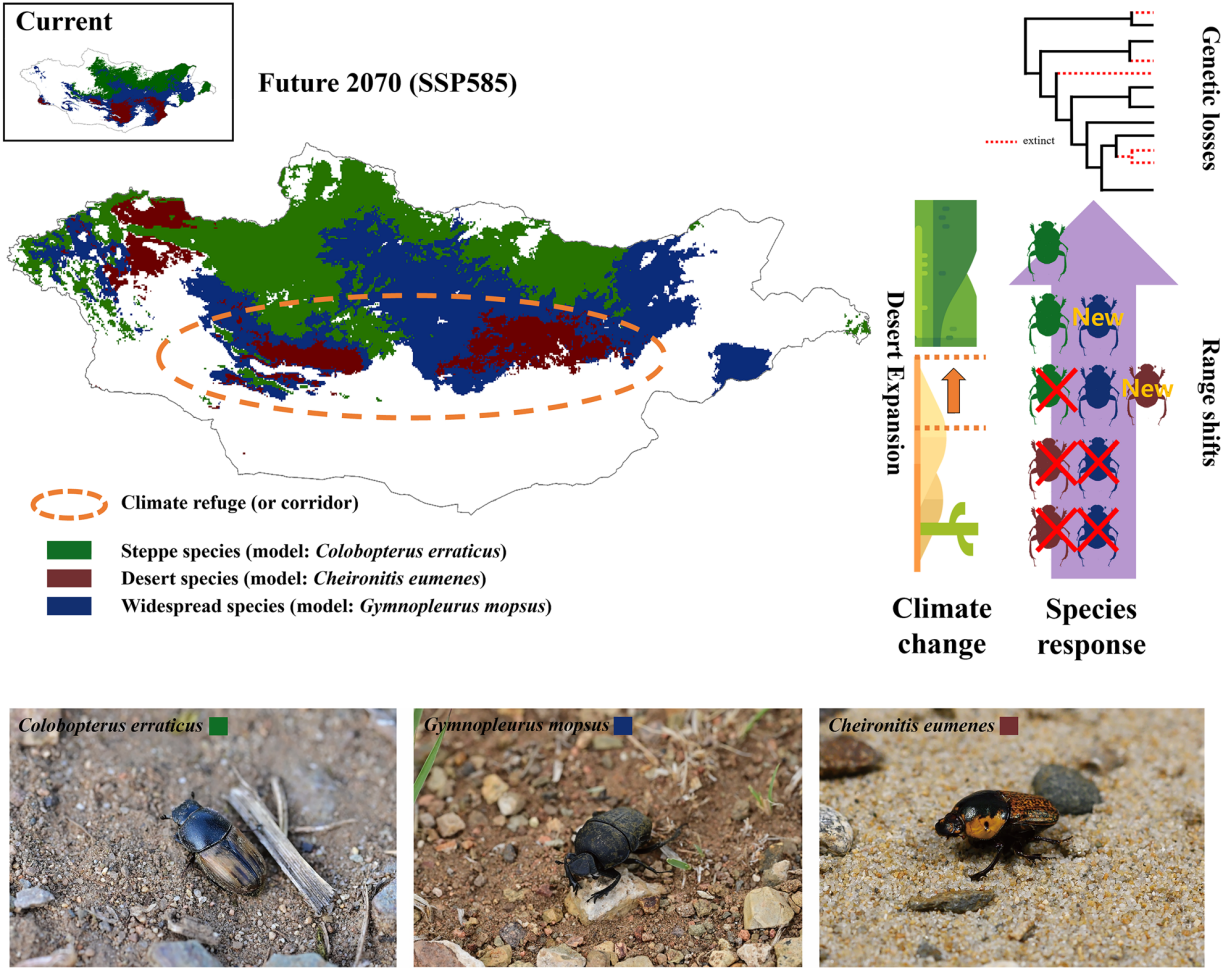
Future distribution of three model dung beetles in 2070 under the SSP585 scenario with schematic illustration of climate change and species response in the Gobi Desert and Mongolian Steppe. The map of distribution model was modified using ArcGIS 10.8 (www.esri.com)^67^.

As a result of climate change, the distribution of steppe-inhabiting dung beetles was expected to shift toward the mountainous steppe. In contrast, the distributions of desert-inhabiting dung beetles were expected to shift toward the central region and to expand northward toward the Gobi Desert (Fig. 5). However, contrary to our expectations, the desert-inhabiting species *C. eumenes* experienced the most severe range contraction. This suggests that the modeled climate change may have exceeded the thermal and drought tolerance of dung beetles in the Gobi Desert. Widespread dung beetles were predicted to lose their current habitat in the southern desert region and to expand their distribution into the northern-central region, which was predicted to develop into a more suitable habitat (i.e., semi-desert habitat). Therefore, it is likely that the central region of Mongolia, which is an intermediate region between desert and steppe, will become a key region for dung beetle diversity conservation over the next 50 years (Fig. 5). National parks in central Mongolia, such as Hustai, Gorkhi-Terelj, and Khugu Tarna, serve as climate refuges and corridors for Mongolian dung beetles. These national parks will likely become the leading edge of the range-shifting populations of *C. eumenes* and the trailing edge (or refuge) and center (or corridor) populations of *C. erraticus* and *G. mopsus,* respectively. Therefore, conservation management in the central region of Mongolia is crucial for conserving biodiversity of Mongolian dung beetles.

In conclusion, we provide novel insights into the potential range shifts and genetic diversity losses of dung beetles from the Gobi Desert and Mongolian Steppe in response to climate change. Contrary to general expectations, desert species appear to be the most vulnerable to climate change due to the extensive degradation in the Gobi Desert. Both steppe and widespread species are likely to experience high rates of occupancy turnover and genetic loss, which could reshuffle the dung beetles community. Climate change-induced desertification should be considered an emerging threat to dung beetles in drylands worldwide.

## Materials and methods

### Study species, sampling, and DNA sequencing

Three dung beetle species that represent the typical biogeographic patterns of dung beetles in Mongolia were selected for this study: *Colobopterus erraticus* (Linnaeus, 1758), *Cheironitis eumenes* (Motschulsky, 1859), and *Gymnopleurus mopsus* (Pallas, 1781). These species generally inhabit the steppe, desert, and both biomes, respectively (Fig. 5). Adult dung beetles were collected either directly from dung pats of livestock (horses, cattle, sheep, goats, camels, and yaks) or using pit-fall traps baited with fresh horse dung. Specific preference for dung type was not observed for any species during field sampling. Specimens were preserved in 95% ethyl alcohol for subsequent DNA analysis.

Genomic DNA was extracted from leg and thoracic tissues using a Qiagen DNeasy Blood and Tissue Kit (Qiagen, Germanton, NC, USA) following the manufacturer’s protocol. A fragment of the mitochondrial cytochrome oxidase I gene (*COI*) was amplified using polymerase chain reaction (PCR) in 20 µL reaction volumes using coleopteran-specific primers (forward: C1-J-2183, 5′-CAA CAT TTA TTT TGA TTT TTT GG-3′; reverse: TL2-N-3014, 5′-TCC AAT GCA CTA ATC TGC CAT ATT A-3′)^47^, universal primers (forward: LCO1490, 5′-GGT CAA CAA ATC ATA AAG ATA TTG G-3′; reverse: HCO2198, 5′-TAA ACT TCA GGG TGA CCA AAA AAT CA-3′)^48^, AccuPower PCR PreMix (Bioneer, Daejeon, South Korea), and the following conditions: initial denaturation at 94 ℃ for 3 min; followed by 36 cycles of 94 °C for 30 s, 47–52 °C for 30 s, and 72 °C for 90 s; and a final extension step at 72 °C for 5 min. Amplification was verified using electrophoresis on 1.5% agarose gels and UV visualization. The verified PCR products were purified enzymatically by Exonuclease I and Shrimp Alkaline Phosphatase (New England BioLabs, Ipswich, MA, USA) and then sequenced by Macrogen Inc. (Seoul, South Korea) using an ABI Prism 3130 Genetic Analyzer (Applied Biosystems, Foster City, CA, USA). All sequences were aligned using the Clustal W^49^ algorithm, implemented in MEGA X v.10.2.6^50^, and were checked manually to confirm alignment. Some of *COI* sequences for *G. mopsus* were obtained from GenBank (MF674025–MF674381)^31^. The newly obtained sequences were deposited in GenBank under accession numbers OR363454–OR363626 (*C. erraticus*), OR363219–363370 (*C. eumenes*), and OR364414–OR364436 (*G. mopsus*).

### Genetic diversity and haplotype network analyses

The number of haplotypes (*Nh*), haplotype diversity (*Hd*), and nucleotide diversity (π) of each population were estimated for each species using DnaSP v.6.12.03^51^. Allelic richness (*Ar*) was calculated using the rarefaction method in CONTRIB v.1.025^52^, that corrects for unequal sample sizes among populations. Haplotype networks for each species were constructed using minimum-spanning tree algorithms in HapStar v.0.7^53^ and optimized using PopART v.1.7^54^. Only populations from which at least five individuals (N ≥ 5) were sampled were included in subsequent genetic analyses, except for demographic history and intraspecific phylogenetic analyses.

### Population structure and demographic history analyses

To infer the genetic differentiation and spatial genetic structure of the three species, ARLEQUIN v.3.5^55^ was used to calculate fixation index (*F*_ST_) values for pairwise population comparisons and to conduct hierarchical analysis of molecular variance (AMOVA, 1000 permutations). Each population of the three species was assigned to a geographic or climatic region, depending on the species’ distribution pattern. The *C. erraticus* populations were assigned to (1) two geographic groups: the Khangai Mountains (CER1621, 1808, and 2208) and others (CER1402, 1502, 1626, 1822, 2213, 2218, and 2222) and (2) three geographic groups: North (CER1402 and 2218), Central (CER1502, 1621, 1626, 2208, 2213, and 2222), and West (CER1808 and 1821). The *C. eumenes* populations were assigned to two geographic groups: southern (CEU1608, 1609, 22A4, 22A6, 22A7, and 22A8) and southeastern (CEU1415 and 1713). The *G. mopsus* populations were assigned to (1) two climatic groups: steppe (GM1403, 1628, 1630, and 2217) and desert (GM1417, 1510, 1604, 1605, 1611, and 162b); (2) three geographic groups: North (GM1403 and 2217), Central (GM1628 and 1630), South (GM1417, 1510, 1604, 1605, 1611, and 162b); and (3) four geographic groups based on major road networks: Northcentral (GM1403 and 1630), Northwest (GM1628 and 2217), South (GM1510, 1604, 1605, and 1611), and Southeast (GM1417 and 162b). The total molecular variance was partitioned among the groups (Φ_ct_ = ′′inter-group′′ genetic variation), within the groups (Φ_sc_ = ′′intra-group′′ genetic variation), and within populations regardless of the grouping (Φ_st_ = ′′inter-population′′ genetic variation).

To infer demographic history, the past demographic expansion of the population of the three species was tested Tajima’s *D*^56^, and Fu’s *Fs*^57^ neutrality tests with 1000 random samples in ARLEQUIN v3.5. Mismatch distribution (distribution of pairwise sequence difference) analysis was performed in DnaSP v.6.12.03 and sum of squared deviations (SSD) and raggedness index values were calculated to assess the goodness of fit between the observed and expected distributions of demographic expansion model.

### Intraspecific phylogeny

Phylogenetic trees of the three species were reconstructed using Bayesian inference (BI) and Maximum likelihood (ML). BI trees were reconstructed using Markov Chain Monte Carlo (MCMC) methods, assuming a constant coalescent size for the tree prior, in BEAST v.10.4^58^. The best-fit evolutionary substitution models were selected using the Akaike information criterion (AICc) in jModelTest v.2.1.7^59^ as follows: TrN + I for *C. erraticus* and *C. eumenes*; GTR + G + I for *G. mopsus*. Strict clock model was selected for three species based on calculation of the Bayes factor^58,60^. A *COI* substitution rate of 0.0075 (substitutions/site/million years) was based on previous phylogenetic study on dung beetles^61^. The MCMC run was performed using 10–100 million generations for each species, with sampling every 1000 generations, and the first 10 or 20% of generations were discarded as burn-in (Table S5). The run was examined using Tracer v.1.6^62^ to confirm that stationarity and convergence had been reached (effective sample size [ESS] > 200). The Maximum Clade Credibility (MCC) tree was evaluated using TreeAnnotator v.10.4^58^. ML analyses were performed using IQ-TREE^63^ with 1000 replicates in ultrafast bootstrap^64^. Intraspecific coalescent trees were visualized using FigTree v.1.4.4^65^.

### Species distribution modeling

From 2009 to 2022, field investigations were conducted at 164 sites distributed across Mongolia, ranging from 50° N to 43° N and 91° E to 112° E. Many SDM studies rely on distribution records from open-access databases, such as the Global Biodiversity Information Facility (GBIF). However, such databases often lack data for neglected regions and species, such as dung beetles in the Gobi Desert and Mongolian Steppe. To address this challenge, this study used data collected during our extensive field investigations conducted over the past 14 years (Table S6). Duplicate occurrence records were eliminated and rarefied to 5 km, resulting in 56, 28, and 45 occurrence records for *C. erraticus, C. eumenes,* and *G. mopsus*, respectively.

Bioclimatic variables for modeling (Table S7) were obtained from WorldClim^66^ at a spatial resolution of 30 arcsec (ca. 1 km^2^) using ArcGIS Desktop 10.8^67^. For future climate projections to 2050 and 2070, three different Shared Socioeconomic Pathways (SSPs), including two moderate stabilization scenarios (SSP245 and SSP370) and one high baseline emission scenario (SSP585), were derived from the CMIP6 global climate model (BCC-CSM)^68^. The limitation of food resources is a crucial factor in determining species’ distribution^69^. However, in Mongolia, the tremendous number of livestock widely distributed through nomadic herding provides sufficient food resources across the landscape (National Statistics Office of Mongolia; https://www.en.nso.mn/, accessed on April 08, 2022)^28,41^.

To avoid multicollinearity and overfitting of the variables in the models, a Pearson’s correlation analysis was conducted using the SDM Toolbox v.2.0^70^ to remove highly correlated bioclimatic variables (Pearson’s correlation coefficient >|0.8|; Table S8), and as a result, subsequent modeling was performed using seven variables, namely annual mean temperature (BIO1), mean diurnal range (BIO2), isothermality (BIO3), temperature seasonality (BIO4), annual precipitation (BIO12), precipitation during the driest month (BIO14), and precipitation seasonality (BIO15).

The current and future distributions of the three dung beetles were inferred using maximum entropy modeling^71^. The maximum entropy model (MaxEnt) is one of the most popular SDMs and uses presence-only data to estimate species distribution and habitat suitability^72^. The ′′ENMeval′′ R package^73^ was used to select the best models for each species based on the optimization of two parameters: regularization multiplier (RM) and feature combination (FC). SDMs were generated using tenfold cross-validation with 10,000 background points. The models were optimized using RM values ranging from 0.5 to 4.0 (in increments of 0.5) and six feature combinations (L, LQ, H, LQH, LQHP, and LQHPT), which resulted in a total of 48 parameter combinations of SDMs for each species. The k-fold cross-validation approach was used instead of the delete-one-jackknife approach, which performs better for small sample sizes^74,75^. The optimized SDMs were evaluated using the difference between the training and testing AUCs (AUC_diff_) and 10% training omission rate (OR_10_), which indicate the degree of overfitting, and the Akaike information criterion corrected for small sample sizes (AICc), which reflects model complexity^14,74,76^. The optimal parameter combination for each species was selected based on the lowest delta.AICc value. To maintain a balance between model complexity and predictive accuracy, the values of OR_10_, AUC_diff_, and AUC_test_ were considered collectively^14,77^.

All SDMs were generated using the ′′ENMTools′′ R package^78^, and MaxEnt v.3.4.4^71^.

### Predicting distribution and genetic diversity under climate change scenarios

To simulate the future distributions and genetic diversities of the three dung beetle species under three climatic scenarios (SSP245, SSP370, and SSP585) at two time points (2050 and 2070), the predicted suitability across Mongolia was transformed into binary maps (presence/absence) based on the 10% training presence logistic threshold on the established final SDMs for each species. Changes in distribution were calculated as the difference in range size between current distributions and those predicted at each time point under each climatic scenario. We assumed that only populations occurring in areas with suitability greater than the present threshold would persist and, thus, contribute to the gene pool of subsequent generations^39^. The number of haplotypes, haplotype diversity, and nucleotide diversity were only calculated for the populations remaining at each time point under each climatic scenario. The loss of haplotypes under different scenarios was visualized in the phylogenetic tree for each species.

### Supplementary Information

Supplementary Information.

## Data Availability

The sequence data that support the findings of this study are openly available in GenBank (https://www.ncbi.nlm.nih.gov/genbank/) under accession number OR363454–OR363626, OR363219–OR363370, and OR364414–OR364436. The geographical coordinates of three model dung beetles occurrences are provided in Table S2.
