# Supplementary material for: Climate change will lead to range shifts and genetic diversity losses of dung beetles in the Gobi Desert and Mongolian Steppe
Source: Sci Rep. 2024 Jul 8;14:15639. doi: 10.1038/s41598-024-66260-1 (PMC11231139; doi:10.1038/s41598-024-66260-1)
Supplement: Supplementary file 1 — Supplementary Information. [file 41598_2024_66260_MOESM1_ESM.docx]

**Supplementary Information**

**Table of Contents:**

| **Title** | **Page** |
| --- | --- |
| Table S1. Pairwise differences of *Colobopterus erraticus* populations in Mongolia. Significant Bonferroni-corrected *F*_ST_ values (*P* < 0.003) are indicated in bold. | 3 |
| Table S2. Pairwise differences of *Cheironitis eumenes* populations in Mongolia. Significant Bonferroni-corrected *F*_ST_ values (*P* < 0.01) are indicated in bold. | 3 |
| Table S3 Pairwise differences of *Gymnopleurus mopsus* populations in Mongolia. Significant Bonferroni-corrected *F*_ST_ values (*P* < 0.0007) are indicated in bold. | 4 |
| Table S4. Predicted habitat suitability for sampled populations of three dung beetle species in Mongolia under current conditions and future climate change scenarios. Populations described below threshold suitability are indicated by bold text and gray highlighting. | 5 |
| Table S5. Data information and setting for Bayesian inference analyses for each target species. | 6 |
| Table S6. Localities, occurrence records of three dung beetles for the present study. | 7 |
| Table S7. The bioclimatic variables examined in this study. | 14 |
| Table S8. Pearson’s correlation coefficients (*r*) of the 19 bioclimatic variables in Mongolia. Highly correlated bioclimatic variables (\|0.8\| < Pearson’s correlation coefficients) are indicated in bold. | 15 |
| Figure S1. Mismatch distribution of nucleotide differences between pairs of *COI* haplotypes. (A) *Colobopterus erraticus*, (B) *Cheironitis eumenes,* and (C) *Gymnopleurus mopsus.* a: the least-squares procedure to fit model mismatch distribution and observed distribution did not converge after 2,000 steps. | 16 |
| Figure S2. The evaluation values of the parameter combinations, implemented in ENMeval. (A) *Colobopterus erraticus*, (B) *Cheironitis eumenes*, and (C) *Gymnopleurus mopsus*. | 17 |
| Figure S3. Distribution and environmental suitability predictions of *Colobopterus erraticus*. (A) Current species distribution model with the occurrence records (white dots), and sites for population genetics (black dots); (B–D) Predicted future distributions in 2050 under the SSP245, SSP370, and SSP585 scenarios, respectively; (E–G) Predicted future distributions in 2070 under the SSP245, SSP370, and SSP585 scenarios, respectively. | 18 |
| Figure S4. Distribution and environmental suitability predictions of *Cheironitis eumenes*. (A) Current species distribution model with the occurrence records (white dots), and sites for population genetics (black dots); (B–D) Predicted future distributions in 2050 under the SSP245, SSP370, and SSP585 scenarios, respectively; (E–G) Predicted future distributions in 2070 under the SSP245, SSP370, and SSP585 scenarios, respectively. | 19 |
| Figure S5. Distribution and environmental suitability predictions of *Gymnopleurus mopsus*. (A) Current species distribution model with the occurrence records (white dots), and sites for population genetics (black dots); (B–D) Predicted future distributions in 2050 under the SSP245, SSP370, and SSP585 scenarios, respectively; (E–G) Predicted future distributions in 2070 under the SSP245, SSP370, and SSP585 scenarios, respectively. | 20 |
| Figure S6. Phylogenetic tree with predicted haplotype loss for *Colobopterus erraticus*. Each terminal nodes (haplotype) is labeled with three pairs of boxes, which represent haplotype retention (black) or loss (white) under three climate change scenarios (SSP245, SSP370, and SSP585; left to right) in 2050 and 2070 (left and right boxes within each pair). | 21 |
| Figure S7. Phylogenetic tree with predicted haplotype loss for *Cheironitis eumenes*. Each terminal nodes (haplotype) is labeled with three pairs of boxes, which represent haplotype retention (black) or loss (white) under three climate change scenarios (SSP245, SSP370, and SSP585; left to right) in 2050 and 2070 (left and right boxes within each pair). | 22 |
| Figure S8. Phylogenetic tree with predicted haplotype loss for *Gymnopleurus mopsus*. Each terminal nodes (haplotype) is labeled with three pairs of boxes, which represent haplotype retention (black) or loss (white) under three climate change scenarios (SSP245, SSP370, and SSP585; left to right) in 2050 and 2070 (left and right boxes within each pair). | 23 |

Table S1. Pairwise differences of *Colobopterus erraticus* populations in Mongolia. Significant Bonferroni-corrected *F*_ST_ values (*P* < 0.003) are indicated in bold.

|  | **CER1402** | **CER1502** | **CER1621** | **CER1626** | **CER1808** | **CER1822** | **CER2208** | **CER2213** | **CER2218** | **CER2222** |
| --- | --- | --- | --- | --- | --- | --- | --- | --- | --- | --- |
| **CER1402** | 0 |  |  |  |  |  |  |  |  |  |
| **CER1502** | 0.00219 | 0 |  |  |  |  |  |  |  |  |
| **CER1621** | 0.03298 | -0.00894 | 0 |  |  |  |  |  |  |  |
| **CER1626** | -0.0406 | -0.06312 | 0.02431 | 0 |  |  |  |  |  |  |
| **CER1808** | 0.29913 | 0.18231 | 0.11409 | 0.24607 | 0 |  |  |  |  |  |
| **CER1822** | 0.03327 | -0.02589 | 0.10962 | -0.03528 | **0.35382** | 0 |  |  |  |  |
| **CER2208** | 0.06879 | -0.01229 | -0.02528 | 0.0433 | 0.09682 | 0.11903 | 0 |  |  |  |
| **CER2213** | -0.00068 | -0.06616 | 0.03418 | -0.01259 | 0.25639 | 0.05087 | 0.03229 | 0 |  |  |
| **CER2218** | -0.0033 | -0.0176 | 0.10031 | -0.06049 | **0.36655** | -0.0228 | 0.11488 | 0.04689 | 0 |  |
| **CER2222** | -0.02854 | -0.03487 | 0.08466 | -0.03733 | **0.35699** | -0.00481 | 0.10412 | 0.01401 | -0.01956 | 0 |

Table S2. Pairwise differences of *Cheironitis eumenes* populations in Mongolia. Significant Bonferroni-corrected *F*_ST_ values (*P* < 0.01) are indicated in bold.

|  | **CEU1609** | **CEU1713** | **CEU1415** | **CEU1608** | **CEU22A4** | **CEU22A6** | **CEU22A7** | **CEU22A8** |
| --- | --- | --- | --- | --- | --- | --- | --- | --- |
| **CEU1609** | 0 |  |  |  |  |  |  |  |
| **CEU1713** | -0.02204 | 0 |  |  |  |  |  |  |
| **CEU1415** | 0.01786 | -0.00056 | 0 |  |  |  |  |  |
| **CEU1608** | -0.08696 | -0.05101 | 0.00000 | 0 |  |  |  |  |
| **CEU22A4** | -0.00918 | 0.00543 | -0.00970 | -0.0664 | 0 |  |  |  |
| **CEU22A6** | -0.00868 | -0.01747 | 0.01198 | -0.0679 | -0.00778 | 0 |  |  |
| **CEU22A7** | 0.03786 | 0.07282 | 0.08092 | -0.04887 | 0.02252 | 0.0427 | 0 |  |
| **CEU22A8** | -0.01493 | -0.00028 | 0.01327 | -0.1031 | -0.00134 | -0.00841 | 0.01014 | 0 |

Table S3 Pairwise differences of *Gymnopleurus mopsus* populations in Mongolia. Significant Bonferroni-corrected *F*_ST_ values (*P* < 0.0007) are indicated in bold.

|  | **GM1403** | **GM1417** | **GM1510** | **GM1604** | **GM1605** | **GM1611** | **GM1628** | **GM1630** | **GM162b** | **GM2217** |
| --- | --- | --- | --- | --- | --- | --- | --- | --- | --- | --- |
| **GM1403** | 0 |  |  |  |  |  |  |  |  |  |
| **GM1417** | 0.00432 | 0 |  |  |  |  |  |  |  |  |
| **GM1510** | -0.00463 | 0.00757 | 0 |  |  |  |  |  |  |  |
| **GM1604** | 0.01149 | 0.00374 | 0.00245 | 0 |  |  |  |  |  |  |
| **GM1605** | 0.03169 | 0.01154 | 0.0149 | -0.01366 | 0 |  |  |  |  |  |
| **GM1611** | 0.02111 | 0.01597 | -0.00372 | -0.00588 | -0.00361 | 0 |  |  |  |  |
| **GM1628** | -0.00816 | 0.01129 | 0.00899 | 0.01998 | **0.04142** | 0.02604 | 0 |  |  |  |
| **GM1630** | 0.00514 | 0.02481 | 0.04457 | **0.06147** | **0.07832** | **0.06026** | 0.00661 | 0 |  |  |
| **GM162b** | 0.0434 | 0.04714 | 0.04987 | 0.01004 | 0.0259 | 0.03031 | 0.04452 | 0.0753 | 0 |  |
| **GM2217** | 0.04217 | 0.03328 | 0.05388 | 0.03378 | 0.06038 | 0.06261 | 0.01399 | 0.06781 | 0.09452 | 0 |

Table S4. Predicted habitat suitability for sampled populations of three dung beetle species in Mongolia under current conditions and future climate change scenarios. Populations described below threshold suitability are indicated by bold text and gray highlighting.

| **Species** | **Sites** | **Current** | **SSP245_2050** | **SSP245_2070** | **SSP370_2050** | **SSP370_2070** | **SSP585_2050** | **SSP585_2070** |
| --- | --- | --- | --- | --- | --- | --- | --- | --- |
| *Colobopterus erraticus* | CER1402 | 0.3021 | **0.2622** | **0.2049** | **0.1857** | **0.0909** | **0.1582** | **0.0867** |
| Threshold: 0.2864 | CER1502 | 0.5333 | 0.5203 | 0.4995 | 0.4274 | 0.2831 | 0.3981 | **0.2616** |
|  | CER16EX2 | **0.2569** | **0.1973** | **0.1418** | **0.1285** | **0.0544** | **0.1049** | **0.0501** |
|  | CER1621 | 0.5447 | 0.4935 | 0.4518 | 0.4357 | **0.2714** | 0.4158 | 0.3030 |
|  | CER1626 | 0.7636 | 0.6535 | 0.5540 | 0.5684 | 0.3087 | 0.4795 | 0.3223 |
|  | CER1808 | **0.2748** | 0.3394 | 0.3830 | 0.3463 | 0.3095 | 0.3399 | **0.2706** |
|  | CER1822 | **0.1076** | **0.0712** | **0.0529** | **0.0496** | **0.0153** | **0.0345** | **0.0125** |
|  | CER1005 | 0.3495 | 0.3916 | 0.4535 | 0.3609 | 0.3514 | 0.3618 | 0.3089 |
|  | CER2208 | 0.7424 | 0.5618 | 0.4211 | 0.4376 | **0.1855** | 0.3435 | **0.1960** |
|  | CER2213 | 0.7889 | 0.6981 | 0.5927 | 0.6022 | 0.3695 | 0.5153 | 0.3592 |
|  | CER2218 | 0.5011 | 0.3782 | 0.2906 | **0.2781** | **0.1346** | **0.2247** | **0.1234** |
|  | CER2222 | 0.6569 | 0.6299 | 0.5757 | 0.5502 | 0.4225 | 0.5089 | 0.3960 |
| *Cheironitis eumenes* | CEU1609 | 0.8742 | 0.4955 | 0.4101 | 0.4938 | **0.0023** | **0.0348** | **0.0044** |
| Threshold: 0.3427 | CEU1713 | 0.7162 | 0.6533 | 0.3977 | 0.3522 | **0.0157** | **0.1056** | **0.0146** |
|  | CEU1415 | 0.6176 | 0.8437 | 0.8127 | 0.7137 | **0.2350** | 0.4247 | **0.2234** |
|  | CEU1706 | 0.6532 | 0.8752 | 0.8495 | 0.7764 | 0.3717 | 0.5185 | **0.3249** |
|  | CEU1607 | 0.7835 | 0.5865 | 0.675 | 0.6356 | **0.0136** | **0.1080** | **0.0200** |
|  | CEU1608 | 0.7873 | 0.4399 | 0.4241 | 0.4532 | **0.0033** | **0.0385** | **0.0063** |
|  | CEU22A4 | 0.6508 | **0.3055** | **0.2850** | **0.2692** | **0.0018** | **0.0218** | **0.0031** |
|  | CEU22A6 | 0.4880 | **0.3377** | 0.4236 | 0.389 | **0.0050** | **0.0419** | **0.0077** |
|  | CEU22A7 | 0.7585 | **0.3107** | 0.4327 | 0.4041 | **0.0056** | **0.0441** | **0.0089** |
|  | CEU22A8 | 0.5468 | **0.2040** | **0.2188** | **0.2012** | **0.0010** | **0.0144** | **0.0011** |
| *Gymnopleurus mopsus* | GM1403 | 0.0935 | **0.1765** | **0.2499** | **0.1828** | 0.3113 | **0.2601** | 0.4082 |
| Threshold: 0.2931 | GM1417 | 0.6223 | 0.4905 | 0.4116 | 0.3549 | **0.1581** | 0.2992 | **0.1120** |
|  | GM1510 | **0.2284** | **0.0531** | **0.0640** | **0.0440** | **0.0114** | **0.0256** | **0.0079** |
|  | GM1604 | 0.7439 | 0.6011 | 0.4726 | 0.5160 | **0.1588** | **0.2873** | **0.1397** |
|  | GM1605 | 0.8063 | 0.6365 | 0.5295 | 0.5797 | **0.1336** | 0.3111 | **0.1552** |
|  | GM1611 | 0.8267 | 0.3967 | 0.3373 | 0.3900 | **0.0958** | **0.2146** | **0.1189** |
|  | GM1628 | 0.3555 | 0.4859 | 0.6108 | 0.5272 | 0.6636 | 0.5358 | 0.5995 |
|  | GM1630 | 0.3046 | 0.4499 | 0.6038 | 0.5038 | 0.6773 | 0.5389 | 0.6345 |
|  | GM162b | 0.5386 | 0.6041 | 0.6936 | 0.5622 | 0.4978 | 0.5600 | 0.3031 |
|  | GM2217 | 0.4167 | 0.6174 | 0.7274 | 0.7122 | 0.6919 | 0.7000 | 0.7317 |

Table S5. Data information and setting for Bayesian inference analyses for each target species.

| **BEAST settings** | ***C. erraticus*** | ***C. eumenes*** | ***G. mopsus*** |
| --- | --- | --- | --- |
| **Number of haplotypes** | 44 | 26 | 135 |
| **Sequence length** | 836 | 789 | 658 |
| **MCMC chain length** | 10 M | 10 M | 100 M |
| **Chain sampling** | 1000 | 1000 | 1000 |
| **Molecular clock** | Strict | Strict | Strict |
| **Substitution model** | TrN + I | TrN + I | GTR + G + I |
| **Population model** | Constant | Constant | Constant |
| **Tree Burn-in (N trees)** | 10% | 10% | 20% |

Table S6. Localities, occurrence records of three dung beetles for the present study.

| **Year** | **Site** | **Locality** | **GPS** | ***G. mopsus*** | ***C. eumenes*** | ***C. erraticus*** | **Remark** |
| --- | --- | --- | --- | --- | --- | --- | --- |
| 2009 | extra | Japanese ger (Shiliin Bulag) Arkhust sum, Töv Aimag | 47°46′34.1″N 107°39′23.5″E | x | x | x |  |
| 2010 | 1 | Egiin gol river, Tunhel sum, Khövsgöl Aimag | 49°56′03.4″N 100°37′39.5″E | x | x | x |  |
| 2010 | 5 | Uurgol river, Tsagaan-uur town, Khövsgöl Aimag | 50°32'15.6"N 101°30'55.6"E | x | x | o |  |
| 2013 | 1 | Kherlen River at 20km Northeast of Mungurmorit, Töv Aimag | 48°18'51.43"N 108°42'37.51"E | x | x | o |  |
| 2013 | 2 | Kherlen River at 24km SouthEast of Mungurmorit (Camp site) | 48°02'26.28"N 108°35'07.01"E | x | x | x |  |
| 2013 | 3 | Forest at 2km Northwest of Mungunmorit, Töv Aimag | 48°13'10.89"N 108°27'06.61"E | x | x | o |  |
| 2014 | 1 | Haraa River at 3.7km South of Bayangol, Selenge Aimag | 48°52'56.24"N 106°06'0.21"E | x | x | o |  |
| 2014 | 2 | Sharyn Tsagaan Nuur at 20km North of Darhan, Selenge Aimag | 49°38'46.26"N 106°01'42.64"E | x | x | o |  |
| 2014 | 3 | Sharyn Stream at 7km West of Javhlant, Selenge Aimag | 49°45'25.43"N 106°9'58.01"E | o | x | x |  |
| 2014 | 4 | Yoroo River at 6km Southeast of Dulaanhaan, Selenge Aimag | 49°52'41.23"N 106°14'41.43"E | x | x | x |  |
| 2014 | 5 | Orkhon River at 1km Southwest of Sukhbaatar Selenge Aimag | 50°13'38.59"N 106°11'53.11"E | x | x | x |  |
| 2014 | 6 | Haraa River at 15km South of Darkhan, Selenge Aimag | 49°23'24.51"N 105°54'0.83"E | o | x | o |  |
| 2014 | 10 | Orkhon River at 100m East of Orkhon, Bulgan Aimag | 48°37'21.07"N 103°32'33.84"E | x | x | x | Not included |
| 2014 | 13 | A0102 Rd at 42km Southeast of Choyr, Gobisumber Aimag | 46°04'44.82"N 108°45'14.14"E | o | x | x | Not included |
| 2014 | 14 | A0102 Rd at 1km Northeast of Dalanjargalan, Dornogobi Aimag | 45°55'34.94"N 109°04'35.02"E | o | x | x |  |
| 2014 | 15 | A0102 Rd at 13km Southeast of Ayrag, Dornogobi Aimag | 45°44'33.65"N 109°28'24.78"E | o | o | x |  |
| 2014 | 17 | A0102 Rd at 30km Northwest of Saynshand, Dornogobi Aimag | 45°09'19.52"N 109°58'0.28"E | o | o | o |  |
| 2015 | 1 | 6.5km West of Zuunmod, Töv Aimag | 47°41'03.29"N 106°52'11.52"E | x | x | x |  |
| 2015 | 2 | 31km South of Zuunmod, Töv Aimag | 47°26'56.36"N 106°46'42.80"E | x | x | o |  |
| 2015 | 3 | 80km South of Zuunmod, Töv Aimag | 47°01'19.21"N 106°35'58.24"E | x | x | x |  |
| 2015 | 4 | 90kim South of Zuunmod, Töv Aimag | 46°55'18.65"N 106°37'45.35"E | x | x | o |  |
| 2015 | 5 | 31km Southwest of Mandalgobi, Dundgobi Aimag | 45°37'29.09"N 105°55'25.53"E | o | x | o |  |
| 2015 | 6 | 2.3km Northeast of Tsogttsetsiy, Umnugobi Aimag | 43°44'49.45"N 105°35'08.99"E | o | o | x |  |
| 2015 | 7 | 8.9km Northeast of Tsogttsetsiy, Umnugobi Aimag | 43°47'45.98"N 105°37'29.07"E | o | o | x |  |
| 2015 | 8 | 29km Northeast of Tsogttsetsiy, Umnugobi Aimag | 43°55'55.46"N 105°47'43.64"E | o | o | x |  |
| 2015 | 9 | 30km west of Manlai, Umnugobi Aimag | 44°02'34.10"N 106°29'54.59"E | o | o | x |  |
| 2015 | 10 | 17km North of Manlai, Umnugobi Aimag | 44°14'16.51"N 106°53'59.43"E | o | x | x |  |
| 2015 | 11 | 38km Northwest of Dalazadgad, Umnugobi Aimag | 43°46'50.54"N 104°47'58.17"E | o | o | x |  |
| 2015 | 12 | 39km Northwest of Dalazadgad, Umnugobi Aimag | 43°47'52.86"N 104°03'39.86"E | x | x | x |  |
| 2015 | 13 | 60km South of Mandal-Ovoo, Umnugobi Aimag | 44°06'28.42"N 104°05'01.16"E | o | o | x |  |
| 2015 | 14 | 41km South of Mandal-Ovoo, Umnugobi Aimag | 44°17'01.47"N 104°05'52.11"E | o | o | x |  |
| 2015 | 15 | 33km Southeast of Sayhan-Ovoo, Dundgobi Aimag | 45°12'53.78"N 104°07'50.87"E | o | o | x |  |
| 2015 | 17 | 29km Northeast of Arvayheer, Ovorkhangai Aimag | 46°30'13.13"N 102°55'46.66"E | x | x | x |  |
| 2015 | 18 | 13km Northwest of Yesonzuyl, Ovorkhangai, Aimag | 46°51'11.91"N 103°23'30.75"E | o | x | o | Not included |
| 2015 | 19 | 70km East of Harhorin, Bulgan Aimag | 47°21'05.83"N 103°43'18.32"E | o | x | x |  |
| 2015 | 20 | 40km Southwest of Lun, Töv Aimag | 47°37'01.05"N 104°51'33.94"E | x | x | x |  |
| 2015 | 21 | 42km Southwest of Lun, Töv Aimag | 47°42'34.48"N 105°00'46.50"E | x | x | x |  |
| 2015 | 22 | Hustayn Nuruu at 37km West of Altanbulag, Töv Aimag | 47°41'38.36"N 105°54'36.64"E | o | x | x |  |
| 2015 | extra | Elsen Tasarkhai, Bulgan Aimag | 47°19'45.4"N 103°41'55.2"E | x | x | x | Not included |
| 2016 | extra1 | Sharyn gol River, 4km north from the Bredge, Selenge Aimag | 49°45'07.79"N 106°09'50.15"E | o | x | x | Not included |
| 2016 | extra2 | Shargyn gol River, 250km North from Ulaanbaatar | 50°05'58.4"N 106°12'47.7"E | x | x | o |  |
| 2016 | 1a | 18km North of Choyr, Gobisumber Aimag | 46°30'28.10"N 108°19'27.79"E | o | x | x |  |
| 2016 | 2b | 38km South of Choyr, Gobisumber Aimag | 46°06'26.39"N 108°43'20.88"E | o | x | x |  |
| 2016 | 1 | 60km North of Delgertsogt Töv Aimag | 46°40'46.98"N 106°31'57.83"E | x | x | x |  |
| 2016 | 2 | 4km South of Luus, Dundgobi Aimag | 45°28'13.19"N 105°44'34.65"E | o | x | x |  |
| 2016 | 3 | 30km South of Khuld, Dundgobi Aimag | 44°56'03.02"N 105°33'01.99"E | o | x | x | Not included |
| 2016 | 4 | 35km South of Khuld, Dundgobi Aimag | 44°54'47.99"N 105°32'37.74"E | o | x | x |  |
| 2016 | 5 | 35km Northeast of Dalanzadgad, Umnugobi Aimag | 43°46'28.95"N 104°47'29.96"E | o | o | x |  |
| 2016 | 6 | 2km South of Dalanzadgad, Umnugobi Aimag | 43°33'07.87"N 104°25'16.94"E | x | x | x |  |
| 2016 | 7 | 20km North of Khankhongor, Umnugobi Aimag | 43°56'06.02"N 104°26'55.70"E | o | o | x |  |
| 2016 | 8 | 60km Southeast of Mandal-Ovoo, Umnugobi Aimag | 44°10'41.30"N 104°22'13.87"E | o | o | x |  |
| 2016 | 9 | 55km South of Mandal-Ovoo, Umnugobi Aimag | 44°09'38.65"N 104°05'29.37"E | o | o | x |  |
| 2016 | 10 | 30km South of Mandal-Ovoo, Umnugobi Aimag | 44°22'05.71"N 104°04'34.01"E | o | x | x | Not included |
| 2016 | 11 | 30km South of Mandal-Ovoo, Umnugobi Aimag | 44°22'33.54"N 104°02'32.60"E | o | o | x |  |
| 2016 | 12 | 60km North of Mandal-Ovoo, Umnugobi Aimag | 45°12'28.48"N 104°07'34.94"E | o | x | x | Not included |
| 2016 | 14 | 25km Northwest of Saikhan-Ovoo, Dundgobi Aimag | 45°35'48.83"N 103°47'23.32"E | o | x | x |  |
| 2016 | 15 | 15km East of Bayangol, Ovorkhangai Aimag | 45°50'05.94"N 103°39'16.73"E | o | x | o |  |
| 2016 | 16 | 10km Northwest of Bayangol, Ovorkhangai Aimag | 45°52'54.36"N 103°22'23.27"E | o | x | x |  |
| 2016 | 17 | 3km South of Khairkhandulaan, Ovorkhangai Aimag | 45°55'57.10"N 102°04'29.00"E | x | x | o |  |
| 2016 | 18 | 20km South of Khairkhandulaan, Ovorkhangai Aimag | 45°48'30.32"N 101°58'25.29"E | x | x | x |  |
| 2016 | 19 | Nariintell, Ovorkhangai Aimag | 45°57'52.67"N 101°28'04.78"E | x | x | o |  |
| 2016 | 20 | 5km North of Ulziit, Bayanhongor Aimag | 46°05'46.04"N 100°47'40.62"E | x | x | x |  |
| 2016 | 21 | 5km South of Endenetsogt, Bayanhongor Aimag | 46°23'15.85"N 100°48'48.63"E | x | x | o |  |
| 2016 | 22 | 20km North of Endenetsogt, Bayanhongor Aimag | 46°35'54.25"N 100°54'28.60"E | x | x | o |  |
| 2016 | 23 | Near the Arkhangai, Arkhangai Aimag | 46°45'35.47"N 100°55'43.00"E | x | x | o |  |
| 2016 | 25 | 25km East of Tsetserleg, Arkhangai Aimag | 47°27'34.40"N 101°45'27.49"E | x | x | x | Not included |
| 2016 | 26 | 20km west of Khotont, Arkhangai Aimag | 47°23'07.53"N 102°12'57.64"E | x | x | o |  |
| 2016 | 27 | 2km Northwest of Khotont, Arhangai Aimag | 47°22'26.29"N 102°26'54.83"E | x | x | o |  |
| 2016 | 28 | 15km West of Ulaanshiveet, Orkhon Aimag | 47°21'02.14"N 103°44'06.94"E | o | x | o |  |
| 2016 | 30 | Khustai National Park, Töv Aimag | 47°44'27.8"N 105°51'58.7"E | o | x | x |  |
| 2017 | 1 | 92km Northwest from Choyr, Töv Aimag | 47°1'13.41"N 107°39'24.25"E | x | x | x |  |
| 2017 | 2 | 32km Northeast from Choyr, Gobisumber Aimag | 46°37'30.40"N 108°13'13.24"E | x | x | x |  |
| 2017 | 3 | 25km Southeast from Choyr, Gobisumber Aimag | 46°12'10.97"N 108°38'31.51"E | o | x | x |  |
| 2017 | 4 | 50km Southeast from Choyr, Dorngobi Aimag | 46°0'11.87"N 108°50'7.61"E | o | x | x |  |
| 2017 | 5 | 77km southeast from Choyr, Dorngobi Aimag | 45°54'15.35"N 109°10'31.82"E | x | o | x |  |
| 2017 | 6 | 91km Southeast from Choyr, Dorngobi Aimag | 45°49'9.25"N 109°18'11.57"E | x | o | x |  |
| 2017 | 7 | 30km North from Saynshand, Dorngobi Aimag | 45°9'0.43"N 109°58'8.16"E | x | o | x | Not included |
| 2017 | 8 | 27km North from Saynshand, Dorngobi Aimag | 45°7'43.04"N 109°59'49.87"E | o | o | x | Not included |
| 2017 | 10 | 20km North from Saynshand, Dorngobi Aimag | 45°5'28.19"N 110°8'0.08"E | o | o | x |  |
| 2017 | 12 | 23km North from Saynshand, Dorngobi Aimag | 45°5'42.90"N 110°02'5.12"E | o | o | x |  |
| 2017 | 13 | 34km North from Saynshand, Dorngobi Aimag | 45°13'28.06"N 110°8'46.33"E | o | o | x |  |
| 2017 | 14 | 82km NorthEast from Saynshand, Dorngobi Aimag | 45°33'17.38"N 110°40'14.96"E | x | x | x |  |
| 2017 | 16 | 9km East from Delgereh, Dorngobi Aimag | 45°47'28.86"N 111°20'26.57"E | x | x | x |  |
| 2017 | 17 | 26km East from Delgerech, Dorngobi Aimag | 45°47'37.50"N 111°33'13.80"E | x | o | x |  |
| 2017 | 18 | 23km West from Bayandelger , Sukhbaatar Aimag | 45°45'11.03"N 112°6'6.99"E | x | o | x |  |
| 2017 | 19 | 18km West from Bayandelger , Sukhbaatar Aimag | 45°51'10.60"N 112°32'44.71"E | x | x | x |  |
| 2017 | 20 | 43km NorthEast from Bayandelger, Sukhbaatar Aimag | 46°01'5.59"N 112°45'54.88"E | x | x | x |  |
| 2017 | 21 | 23km West from Baruun-Urt, Sukhbaatar Aimag | 46°43'59.55"N 112°59'18.25"E | x | x | o |  |
| 2017 | 22 | 60km East from Ondorkhaan Khentii Aimag | 47°11'11.97"N 111°25'33.11"E | x | x | x |  |
| 2017 | 23 | 17km East from Ondorkhaan Khentii Aimag | 47°16'49.36"N 110°52'44.80"E | x | x | o |  |
| 2017 | 24 | Khelen River near Ondorkhaan, Khentii Aimag | 47°18'32.96"N 110°39'43.64"E | x | x | x |  |
| 2017 | 25 | 2km South from Ondorkhaan, Khentii Aimag | 47°18'19.69"N 110°39'21.81"E | x | x | x | Not included |
| 2017 | 26 | 55km West from Ondorkhaan, Khentii Aimag | 47°25'01.82"N 109°55'54.18"E | x | x | x |  |
| 2017 | 27 | 121km West from Ondorkhaan, Khentii Aimag | 47°39'34.72"N 109°08'0.35"E | x | x | o |  |
| 2017 | 28 | Kherlen River, 120km East from Ulaanbaatar, Khentii Aimag | 47°41'45.29"N 108°28'30.24"E | x | x | x |  |
| 2017 | 29 | 103km East from Ulaanbaatar, Töv Aimag | 47°43'38.93"N 108°15'57.73"E | x | x | x |  |
| 2018 | 2 | 64km NorthEast from Uliastai, Zavkhan Aimag | 48°04'41.09"N 97°32'36.64"E | x | x | o |  |
| 2018 | 3 | 64km NorthEast from Uliastai, Zavkhan Aimag | 48°06'19.70"N 97°27'02.21"E | x | x | x |  |
| 2018 | 4 | South from Ider town, Zavkhan Aimag | 48°11'5.84"N 97°22'31.02"E | x | x | x |  |
| 2018 | 5 | 39km East of Uliastai, Zavkhan Aimag | 47°39'26.07"N 97°20'37.14"E | x | x | x |  |
| 2018 | 6 | 36km East of Uliastai, Zavkhan Aimag | 47°40'16.44"N 97°18'53.02"E | x | x | x | Not included |
| 2018 | 7 | Dayan Camp, 31km East of Uliastai, Zavkhan Aimag | 47°41'22.65"N 97°15'30.93"E | x | x | x |  |
| 2018 | 8 | 20km East of Uliastai, Zavkhan Aimag | 47°38'48.10"N 97°03'49.16"E | x | x | o |  |
| 2018 | 9 | 5km East of Uliastai, Zavkhan Aimag | 47°44'04.96"N 96°55'03.12"E | x | x | o |  |
| 2018 | 10 | 25km Northeast of Durvuljin, Zavkhan Aimag | 47°46'27.85"N 95°17'38.01"E | x | x | x |  |
| 2018 | 11 | 20km NorthEast of Durvuljin, Zavkhan Aimag | 47°44'38.66"N 95°12'57.97"E | x | x | x |  |
| 2018 | 12 | 11km North of Durvuljin, Zavkhan Aimag | 47°44'47.12"N 94°58'11.21"E | x | x | x |  |
| 2018 | 13 | 10km South of Urgamal, Zavkhan Aimag | 48°26'6.16"N 94°20'1.96"E | x | x | x |  |
| 2018 | 14 | 40km NW of Urgamal, Uvs Aimag | 48°49'29.59"N 93°57'45.01"E | x | x | x |  |
| 2018 | 15 | 90km East of Naranbulag, Uvs Aimag | 49°12'47.19"N 93°46'10.09"E | x | x | x |  |
| 2018 | 20 | 15km North of Tugrug, Khovd Aimag | 47°33'28.71"N 92°10'11.17"E | x | x | x |  |
| 2018 | 21 | 30km South of Tugrug, Khovd Aimag | 47°09'44.75"N 92°14'49.45"E | x | x | x |  |
| 2018 | 22 | Khovd, Khovd Aimag | 48°00'59.70"N 91°37'15.25"E | x | x | o |  |
| 2019 | 2 | Northeast of Nalaikh, Ulaanbaatar | 47°51'29.78"N 107°25'17.93"E | x | x | o |  |
| 2019 | 3 | Teleji National Park, Töv Aimag | 47°59'28.99"N 107°27'38.83"E | x | x | o |  |
| 2019 | 4 | 70km North of Mandalgobi, Dundgobi Aimag | 46°29'18.11"N 106°31'0.78"E | x | x | o |  |
| 2019 | 6 | Near the Khuld town, Dundgobi Aimag | 45°12'32.22"N 105°34'17.60"E | o | x | x |  |
| 2019 | 7 | Near the Khuld town, Dundgobi Aimag | 45°08'47.74"N 105°33'50.85"E | o | o | x |  |
| 2019 | 8 | Near the Khuld town, Dundgobi Aimag | 45°03'41.25"N 105°34'42.18"E | o | o | x |  |
| 2019 | 9 | Near the Khuld town, Dundgobi Aimag | 44°51'35.8"N 105°31'00.8"E | o | o | x |  |
| 2019 | 13 | 18km North from Dalanzadgad, Umnugobi Aimag | 43°39'37.91"N 104°35'40.58"E | o | x | x |  |
| 2019 | 15 | 57km North from Dalanzadgad, Umnugobi Aimag | 43°55'56.6"N 104°57'12.3"E | o | o | x | Not included |
| 2022 | 1 | 25km East of Harhorin, Ovorkhangai Aimag | 47°14'59.0"N 103°08'41.1"E | x | x | o |  |
| 2022 | 2 | 9km Southeast of Harhorin, Ovorkhangai Aimag | 47°08'51.6"N 102°55'18.2"E | x | x | o |  |
| 2022 | 3 | 5km East of Hotont, Arkhangai Aimag | 47°20'40.9"N 102°31'49.1"E | x | x | o |  |
| 2022 | 4 | 37km South of Tsenkher, Arkhangai Aimag | 47°6'34.20"N 101°46'34.22"E | x | x | o |  |
| 2022 | 5 | 13km South of Tsenkher, Arkhangai Aimag | 47°20'01.86"N 101°47'05.20"E | x | x | o |  |
| 2022 | 6 | Tsenkher, Arkhangai Aimag | 47°27'06.60"N 101°44'54.18"E | x | x | o |  |
| 2022 | 7 | 37km Southwest of Tsetserleg, Arkhangai Aimag | 47°14'45.03"N 101°08'33.97"E | x | x | o |  |
| 2022 | 8 | Ikh-Tamir, Arkhangai Aimag | 47°35'17.73"N 101°12'51.48"E | x | x | o |  |
| 2022 | 9 | 35km Northwest of Ikh-Tamir, Arkhangai Aimag | 47°49'29.9"N 100°53'18.78"E | x | x | o |  |
| 2022 | 10 | 40km North of Ikh-Tamir, Arkhangai Aimag | 47°56'17.28"N 101°1'0.44"E | x | x | o |  |
| 2022 | 11 | 50km North of Ikh-Tamir, Arkhangai Aimag | 48°2'47.72"N 101°5'18.29"E | x | x | o |  |
| 2022 | 12 | 42km South of Erdenemandal, Arkhangai Aimag | 48°10'36.51"N 101°11'25.19"E | x | x | x |  |
| 2022 | 13 | 16km South of Erdenemandal, Arkhangai Aimag | 48°23'44.08"N 101°18'46.40"E | x | x | o |  |
| 2022 | NS1 | Erdenemandal, Arkhangai Aimag | 48°31'53.21"N 101°22'18.29"E | x | x | o | Not included |
| 2022 | 14 | 5km North of Erdenemandal, Arkhangai Aimag | 48°34'9.10"N 101°23'54.14"E | x | x | o |  |
| 2022 | 15 | 3km North of Rashaant, Khövsgöl Aimag | 49°9'8.71"N 101°26'24.88"E | x | x | o |  |
| 2022 | 16 | 20km East of Tosontsengel, Khövsgöl Aimag | 49°23'32.17"N 101°7'52.39"E | x | x | o |  |
| 2022 | 17 | 5km East of Ikh-Uul, Khövsgöl Aimag | 49°27'32.61"N 101°32'20.78"E | o | x | x |  |
| 2022 | 18 | 3km South of Tarialan, Khövsgöl Aimag | 49°35'31.79"N 101°59'14.78"E | x | x | o |  |
| 2022 | 19 | 18km Northwest of Khutag-Undur, Bulgan Aimag | 49°29'34.81"N 102°29'54.52"E | x | x | o |  |
| 2022 | 20 | 10km East of Khutag-Undur, Bulgan Aimag | 49°22'39.86"N 102°50'23.48"E | x | x | o |  |
| 2022 | 21 | 30km East of Khutag-Undur, Bulgan Aimag | 49°19'28.74"N 103°4'51.46"E | x | x | o |  |
| 2022 | 22 | 20km South of Unit, Bulgan Aimag | 48°57'47.33"N 102°47'27.31"E | x | x | o |  |
| 2022 | 23 | 32km Southeast of Unit, Bulgan Aimag | 48°58'44.51"N 103°10'0.35"E | x | x | x |  |
| 2022 | 24 | 27km Northwest of Bulgan, Bulgan Aimag | 48°59'39.95"N 103°15'13.37"E | x | x | x |  |
| 2022 | 25 | Orkhon, Bulgan Aimag | 48°37'10.83"N 103°32'30.12"E | o | x | o |  |
| 2022 | 26 | 20km East of Erdenet, Orkhon Aimag | 49°4'30.75"N 104°19'47.09"E | x | x | o |  |
| 2022 | 27 | Bulgan, Bulgan Aimag | 48°48'21.45"N 103°32'11.28"E | x | x | x |  |
| 2022 | 28 | 20km Southeast of Orkhon, Bulgan Aimag | 48°28'25.28"N 103°41'5.66"E | x | x | o |  |
| 2022 | 29 | 54km South of Ulaanbaatar, Töv Aimag | 47°11'8.10"N 106°38'32.88"E | x | x | o |  |
| 2022 | A1 | 22km Westsouth of Mandalgobi, Dundgobi Aimag | 45°40'15.3"N 106°00'08.02"E | x | x | x | Not included |
| 2022 | A2 | 10km North of Khuld, Dundgobi Aimag | 45°18'9.81"N 105°37'2.66"E | x | x | x | Not included |
| 2022 | A3 | 40km South of Khuld, Dundgobi Aimag | 44°51'11.68"N 105°30'23.33"E | x | x | x | Not included |
| 2022 | A4 | 14km South of Tsogt-Ovoo, Umnugobi Aimag | 44°21'18.26"N 105°15'26.49"E | o | o | x |  |
| 2022 | A5 | 45km Southwest of Tsogt-Ovoo, Umnugobi Aimag | 44°4'41.93"N 105°01'58.72"E | x | x | x | Not included |
| 2022 | A6 | 63km Southwest of Tsogt-Ovoo, Umnugobi Aimag | 43°56'56.6"N 104°57'41.0"E | o | o | x |  |
| 2022 | A7 | 54km Northeast of Dalanzadgad, Umnugobi Aimag | 43°54'51.3"N 104°55'14.4"E | x | o | x |  |
| 2022 | A8 | 23km Northeast of Dalanzadgad, Umnugobi Aimag | 43°41'12.7"N 104°39'42.2"E | o | o | x |  |
| 2022 | A9 | 20km North of Tsogt-Ovoo, Umnugobi Aimag | 44°36'04.9"N 105°22'22.8"E | x | x | x | Not included |
| 2022 | A10 | 12km Westsouth of Mandalgobi, Dundgobi Aimag | 45°42'11.4"N 106°06'13.9"E | x | x | x | Not included |
| 2022 | A13 | Teleji National Park, Töv Aimag | 48°02'13.89"N 107°38'20.06"E | x | x | o |  |
| 2022 | A16 | Northeast of Nalaikh, Ulaanbaatar | 47°47'36.94"N 107°19'18.53"E | x | x | o |  |
| **Total** | | | | 45 | 28 | 56 |  |

Table S7. The bioclimatic variables examined in this study.

| **Variable** | **Definition** |
| --- | --- |
| BIO1 | Annual mean temperature |
| BIO2 | Mean diurnal range (max temp – min temp) |
| BIO3 | Isothermality (BIO2/BIO7)*100 |
| BIO4 | Temperature seasonality (standard deviation*100) |
| BIO5 | Max temperature of warmest month |
| BIO6 | Min temperature of coldest month |
| BIO7 | Temperature annual range (BIO5 - BIO6) |
| BIO8 | Mean temperature of wettest quarter |
| BIO9 | Mean temperature of driest quarter |
| BIO10 | Mean temperature of warmest quarter |
| BIO11 | Mean temperature of coldest quarter |
| BIO12 | Annual precipitation |
| BIO13 | Precipitation of wettest month |
| BIO14 | Precipitation of driest month |
| BIO15 | Precipitation seasonality (coefficient of variation) |
| BIO16 | Precipitation of wettest quarter |
| BIO17 | Precipitation of driest quarter |
| BIO18 | Precipitation of warmest quarter |
| BIO19 | Precipitation of coldest quarter |

Table S8. Pearson’s correlation coefficients (*r*) of the 19 bioclimatic variables in Mongolia. Highly correlated bioclimatic variables (|0.8| < Pearson’s correlation coefficients) are indicated in bold.

|  | **BIO1** | **BIO2** | **BIO3** | **BIO4** | **BIO5** | **BIO6** | **BIO7** | **BIO8** | **BIO9** | **BIO10** | **BIO11** | **BIO12** | **BIO13** | **BIO14** | **BIO15** | **BIO16** | **BIO17** | **BIO18** | **BIO19** |
| --- | --- | --- | --- | --- | --- | --- | --- | --- | --- | --- | --- | --- | --- | --- | --- | --- | --- | --- | --- |
| **BIO1** | 1.000 |  |  |  |  |  |  |  |  |  |  |  |  |  |  |  |  |  |  |
| **BIO2** | -0.181 | 1.000 |  |  |  |  |  |  |  |  |  |  |  |  |  |  |  |  |  |
| **BIO3** | -0.277 | 0.500 | 1.000 |  |  |  |  |  |  |  |  |  |  |  |  |  |  |  |  |
| **BIO4** | 0.029 | 0.329 | -0.633 | 1.000 |  |  |  |  |  |  |  |  |  |  |  |  |  |  |  |
| **BIO5** | **0.910** | 0.050 | -0.437 | 0.419 | 1.000 |  |  |  |  |  |  |  |  |  |  |  |  |  |  |
| **BIO6** | **0.823** | -0.437 | 0.035 | -0.515 | 0.535 | 1.000 |  |  |  |  |  |  |  |  |  |  |  |  |  |
| **BIO7** | 0.064 | 0.511 | -0.483 | 0.969 | 0.458 | -0.506 | 1.000 |  |  |  |  |  |  |  |  |  |  |  |  |
| **BIO8** | **0.940** | -0.051 | -0.469 | 0.365 | **0.993** | 0.595 | 0.387 | 1.000 |  |  |  |  |  |  |  |  |  |  |  |
| **BIO9** | **0.864** | -0.269 | -0.026 | -0.316 | 0.655 | **0.896** | -0.273 | 0.698 | 1.000 |  |  |  |  |  |  |  |  |  |  |
| **BIO10** | **0.940** | -0.051 | -0.468 | 0.364 | **0.993** | 0.596 | 0.387 | **1.000** | 0.698 | 1.000 |  |  |  |  |  |  |  |  |  |
| **BIO11** | **0.866** | -0.311 | 0.081 | -0.472 | 0.598 | **0.985** | -0.425 | 0.648 | **0.918** | 0.649 | 1.000 |  |  |  |  |  |  |  |  |
| **BIO12** | -0.660 | 0.434 | 0.393 | 0.034 | -0.555 | -0.598 | 0.063 | -0.596 | -0.649 | -0.596 | -0.593 | 1.000 |  |  |  |  |  |  |  |
| **BIO13** | -0.599 | 0.479 | 0.429 | 0.025 | -0.497 | -0.548 | 0.069 | -0.541 | -0.578 | -0.541 | -0.533 | **0.983** | 1.000 |  |  |  |  |  |  |
| **BIO14** | -0.544 | 0.190 | 0.053 | 0.182 | -0.417 | -0.561 | 0.165 | -0.445 | -0.603 | -0.445 | -0.570 | 0.664 | 0.572 | 1.000 |  |  |  |  |  |
| **BIO15** | 0.183 | 0.335 | 0.268 | -0.019 | 0.206 | 0.154 | 0.048 | 0.179 | 0.220 | 0.179 | 0.185 | 0.199 | 0.343 | -0.361 | 1.000 |  |  |  |  |
| **BIO16** | -0.606 | 0.477 | 0.426 | 0.030 | -0.503 | -0.555 | 0.070 | -0.546 | -0.592 | -0.546 | -0.543 | **0.989** | **0.995** | 0.578 | 0.329 | 1.000 |  |  |  |
| **BIO17** | -0.596 | 0.212 | 0.050 | 0.211 | -0.453 | -0.621 | 0.191 | -0.484 | -0.642 | -0.484 | -0.631 | 0.695 | 0.597 | 0.578 | -0.386 | 0.604 | 1.000 |  |  |
| **BIO18** | -0.606 | 0.477 | 0.426 | 0.030 | -0.503 | -0.555 | 0.070 | -0.546 | -0.592 | -0.546 | -0.543 | **0.989** | **0.995** | 0.578 | 0.329 | **1.000** | 0.604 | 1.000 |  |
| **BIO19** | -0.590 | 0.201 | -0.003 | 0.268 | -0.429 | -0.643 | 0.239 | -0.462 | -0.642 | -0.462 | -0.655 | 0.651 | 0.553 | **0.934** | -0.403 | 0.559 | **0.986** | 0.559 | 1.000 |


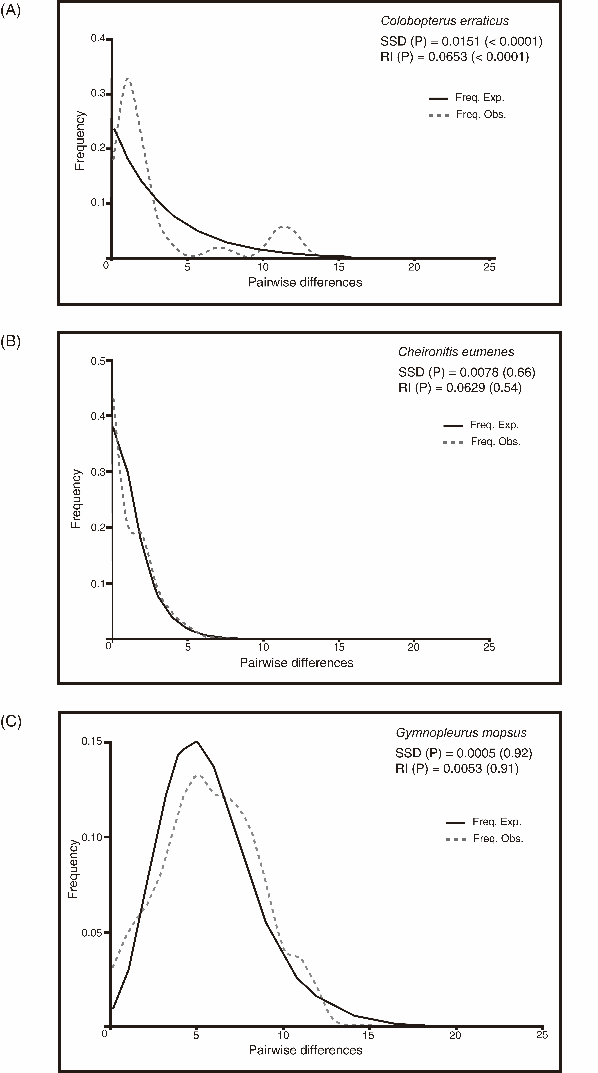
Figure S1. Mismatch distribution of nucleotide differences between pairs of *COI* haplotypes. (A) *Colobopterus erraticus*, (B) *Cheironitis eumenes,* and (C) *Gymnopleurus mopsus.* a: the least-squares procedure to fit model mismatch distribution and observed distribution did not converge after 2,000 steps.


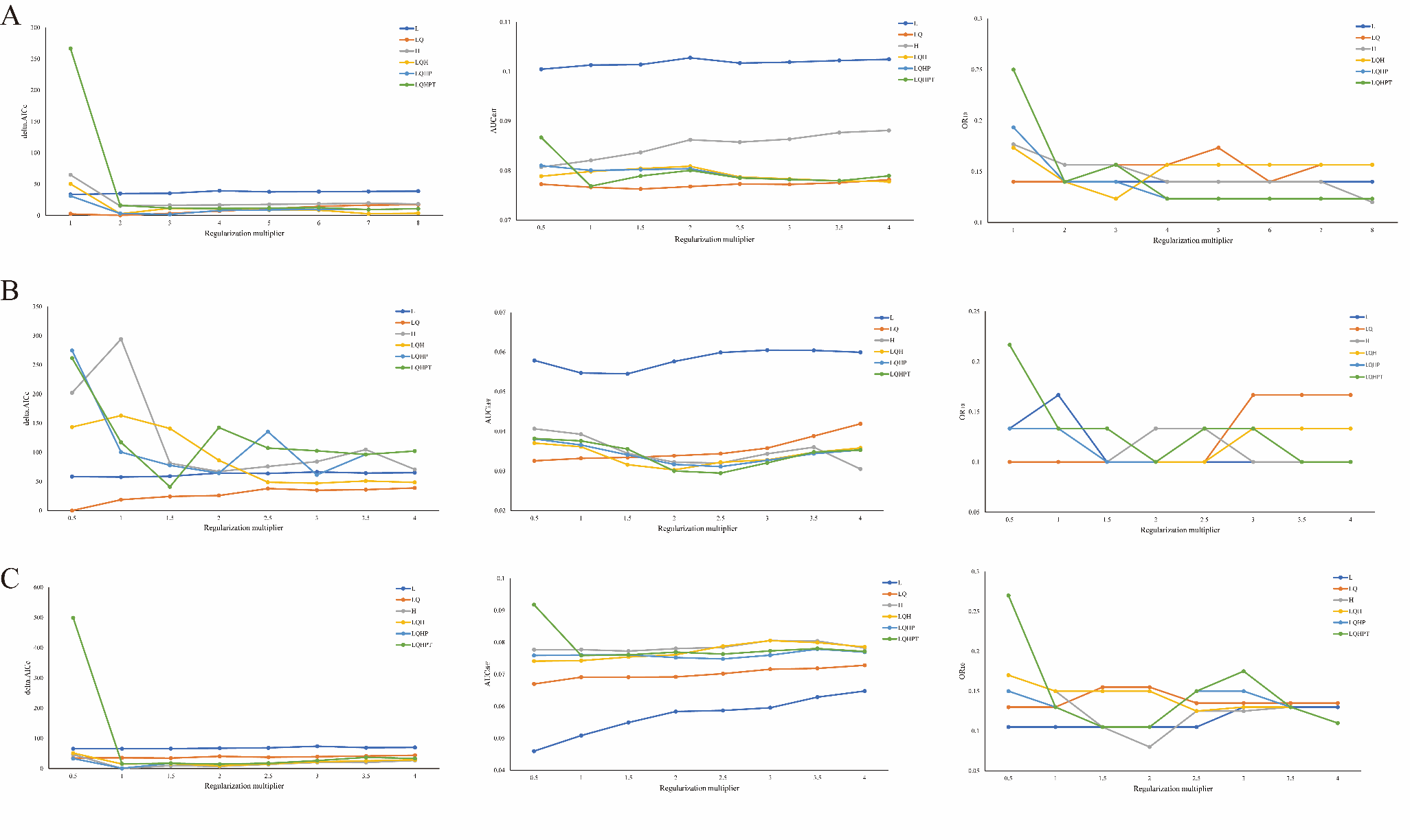


Figure S2. The evaluation values of the parameter combinations, implemented in ENMeval. (A) *Colobopterus erraticus*, (B) *Cheironitis eumenes*, and (C) *Gymnopleurus mopsus*.


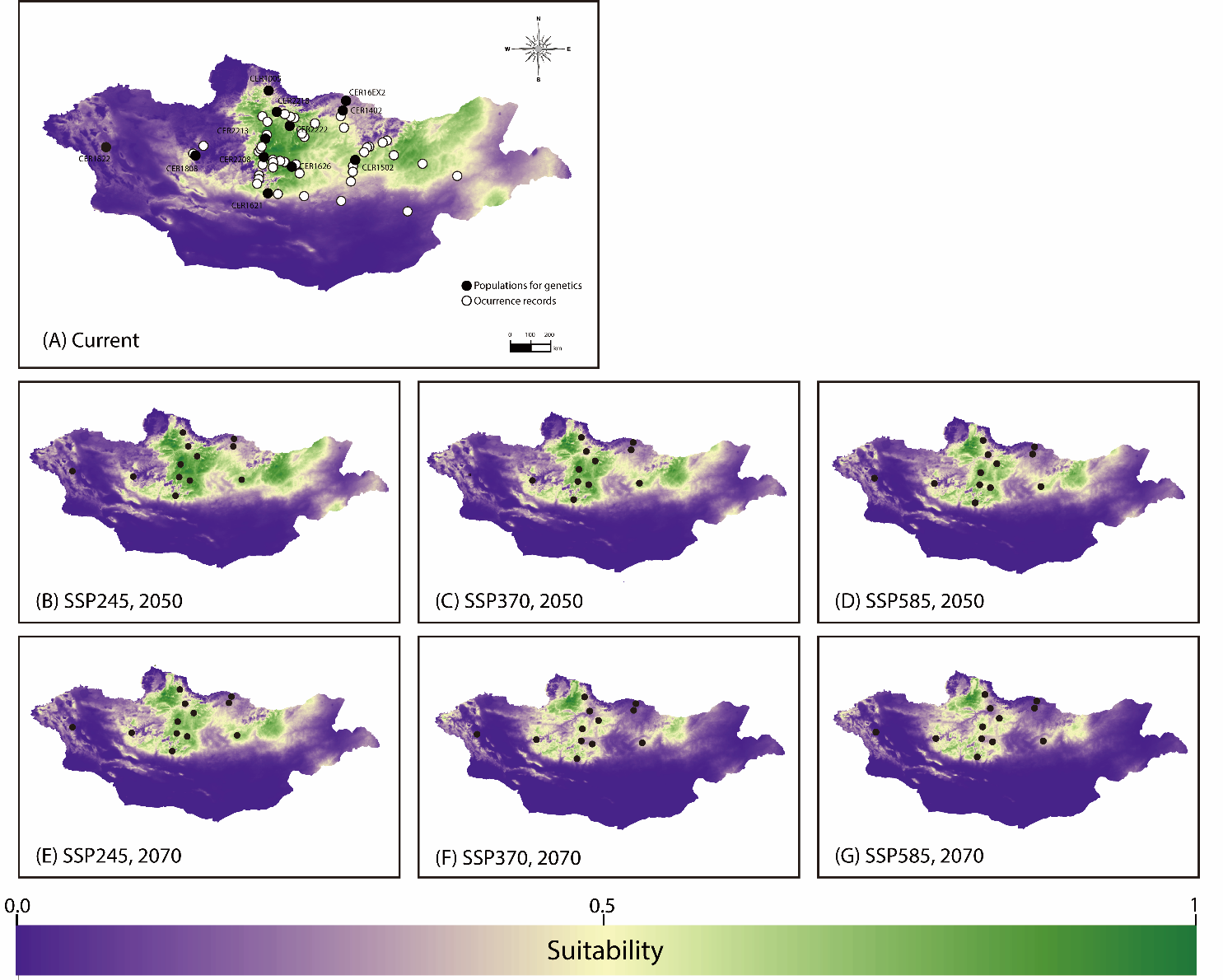


Figure S3. The evaluation values of the parameter combinations, implemented in ENMeval. (A) *Colobopterus erraticus*, (B) *Cheironitis eumenes*, and (C) *Gymnopleurus mopsus*.


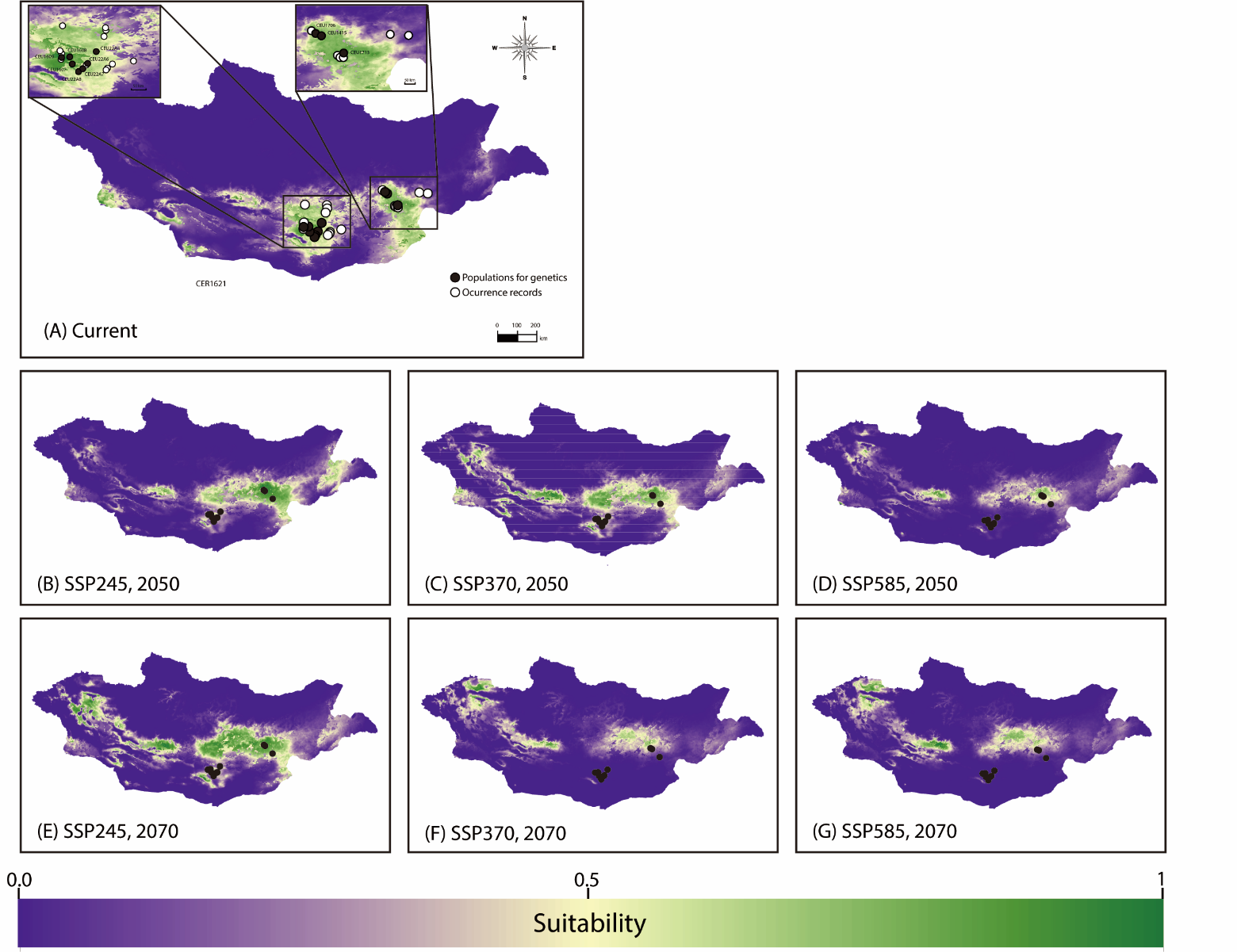


Figure S4. Distribution and environmental suitability predictions of *Cheironitis eumenes*. (A) Current species distribution model with the occurrence records (white dots), and sites for population genetics (black dots); (B–D) Predicted future distributions in 2050 under the SSP245, SSP370, and SSP585 scenarios, respectively; (E–G) Predicted future distributions in 2070 under the SSP245, SSP370, and SSP585 scenarios, respectively.


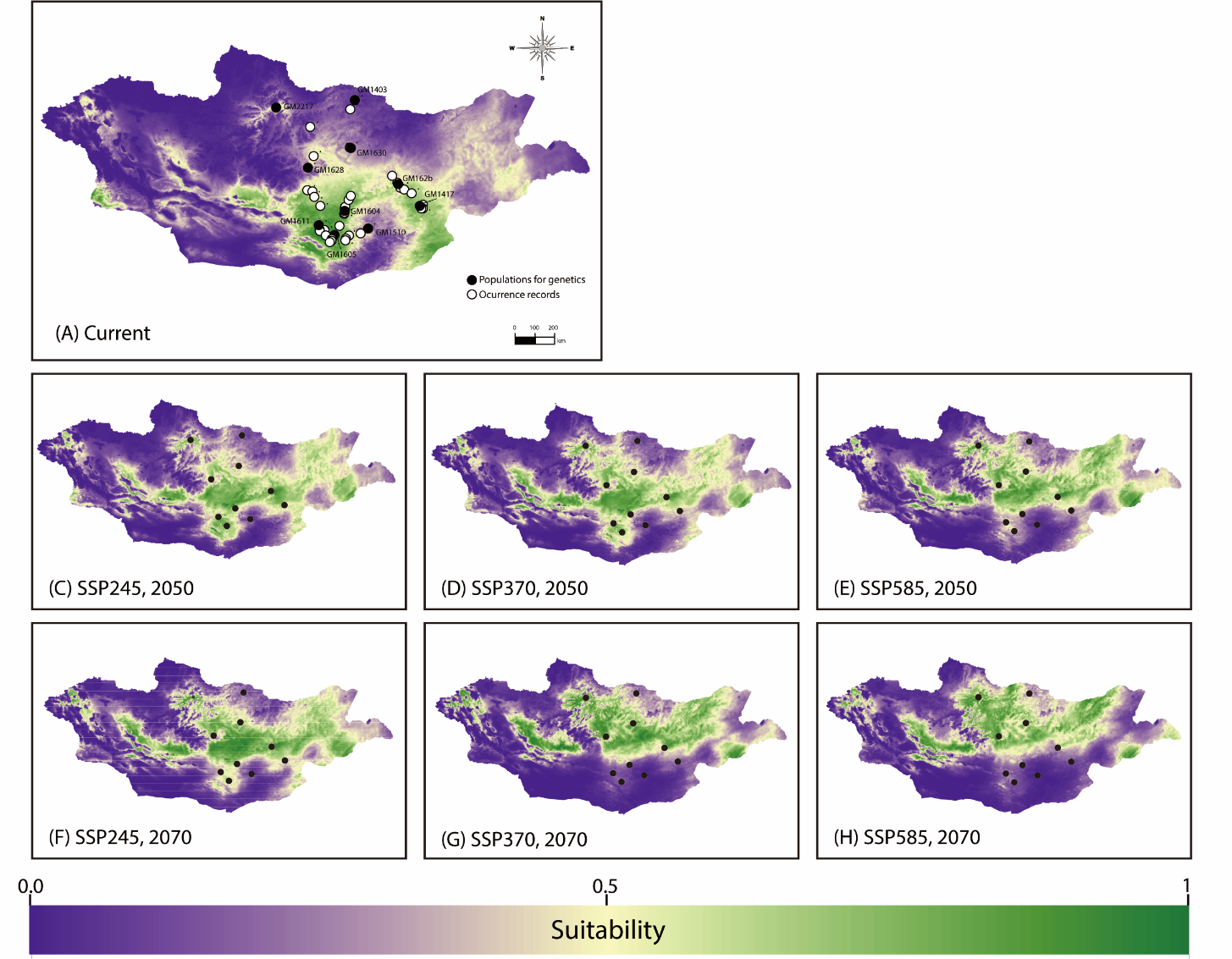


Figure S5. Distribution and environmental suitability predictions of *Gymnopleurus mopsus*. (A) Current species distribution model with the occurrence records (white dots), and sites for population genetics (black dots); (B–D) Predicted future distributions in 2050 under the SSP245, SSP370, and SSP585 scenarios, respectively; (E–G) Predicted future distributions in 2070 under the SSP245, SSP370, and SSP585 scenarios, respectively.


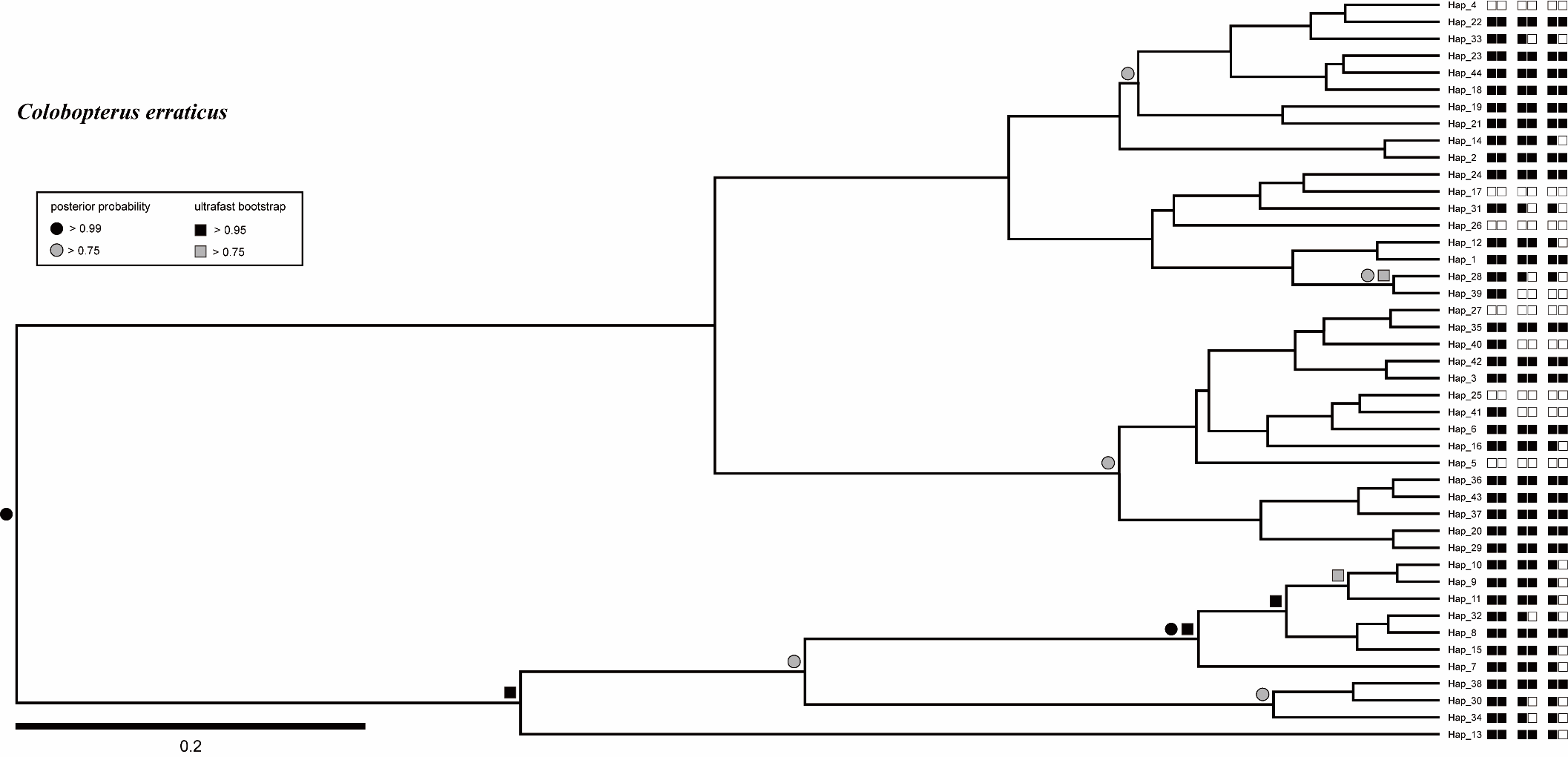


Figure S6. Phylogenetic tree with predicted haplotype loss for *Colobopterus erraticus*. Each terminal nodes (haplotype) is labeled with three pairs of boxes, which represent haplotype retention (black) or loss (white) under three climate change scenarios (SSP245, SSP370, and SSP585; left to right) in 2050 and 2070 (left and right boxes within each pair). Supporting values of posterior probability and ultrafast bootstrap were visualized, and incongruent between Bayesian inference and Maximum likelihood or lower support values ($\leq$ 75) were not displayed.


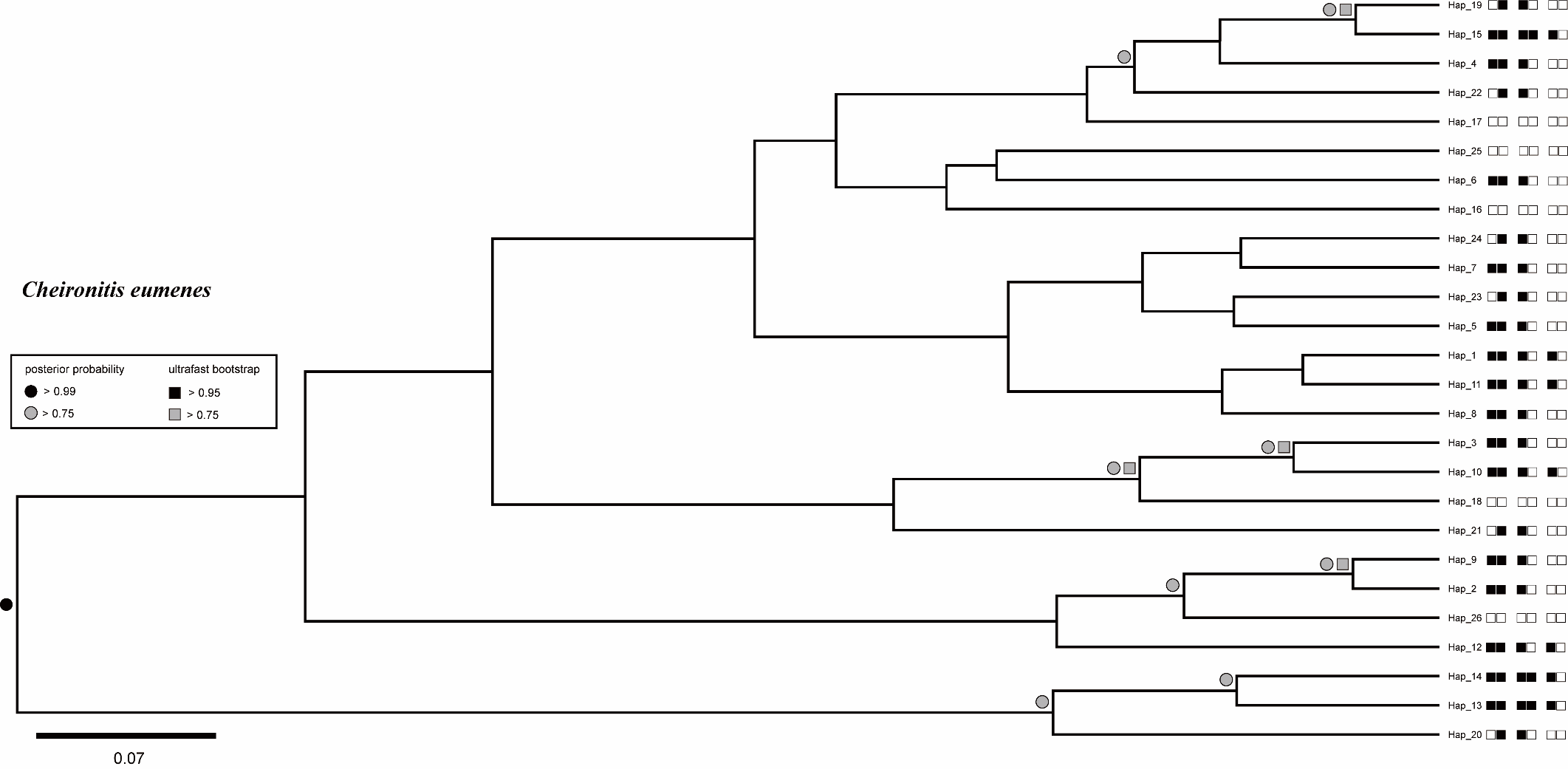


Figure S7. Phylogenetic tree with predicted haplotype loss for *Cheironitis eumenes*. Each terminal nodes (haplotype) is labeled with three pairs of boxes, which represent haplotype retention (black) or loss (white) under three climate change scenarios (SSP245, SSP370, and SSP585; left to right) in 2050 and 2070 (left and right boxes within each pair). Supporting values of posterior probability and ultrafast bootstrap were visualized, and incongruent between Bayesian inference and Maximum likelihood or lower support values ($\leq$ 75) were not displayed.


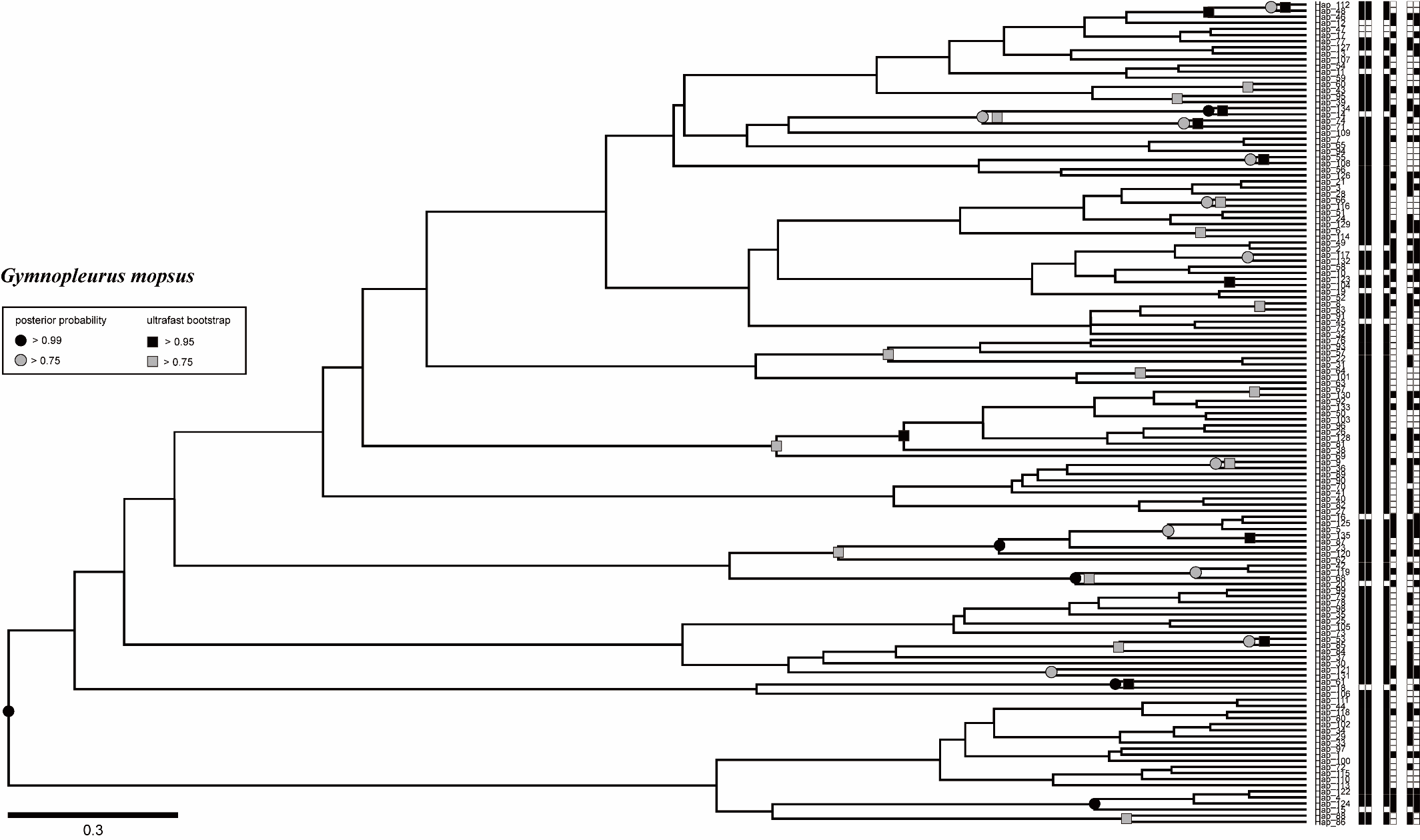


Figure S8. Phylogenetic tree with predicted haplotype loss for *Gymnopleurus mopsus*. Each terminal nodes (haplotype) is labeled with three pairs of boxes, which represent haplotype retention (black) or loss (white) under three climate change scenarios (SSP245, SSP370, and SSP585; left to right) in 2050 and 2070 (left and right boxes within each pair). Supporting values of posterior probability and ultrafast bootstrap were visualized, and incongruent between Bayesian inference and Maximum likelihood or lower support values ($\leq$ 75) were not displayed.
